# Epidemiological evidence for associations between variants in microRNA or biosynthesis genes and lung cancer risk

**DOI:** 10.1002/cam4.2645

**Published:** 2020-01-07

**Authors:** Guanchu Liu, Jie Tian, Chunjian Zuo, Yufu Li, Kui Fu, Huanwen Chen

**Affiliations:** ^1^ Department of Cardiothoracic Surgery The First Affiliated Hospital of Chongqing Medical University Chongqing China; ^2^ Department of Thoracic Surgery The Third Affiliated Hospital of Chongqing Medical University (Gener Hospital) Chongqing China; ^3^ Department of Cardiothoracic Surgery The People's Hospital of Chongqing Tongnan Chongqing China; ^4^ Department of Cardiothoracic Surgery Traditional Chinese Medicine Hospital, Dianjiang Chongqing China

**Keywords:** biosynthesis genes, genetic variant, lung cancer, meta‐analysis, microRNAs

## Abstract

In the past decade, the studies involving single nucleotide polymorphisms (SNPs) in microRNAs (miRNAs) with lung cancer (LC) risk have been performed, however, these results are inconsistent, and a systematic research synopsis has not been performed yet. Therefore, we attempted to perform comprehensive meta‐analyses to assess the relationships between SNPs in miRNAs or biosynthesis genes and LC risk and further evaluate the epidemiological credibility of these significant associations. We used PubMed, Medline, and Web of Science to search for relevant articles published before 30 May 2019 that assessed relationships between SNPs in miRNAs or biosynthesis genes and LC risk. The cumulative epidemiological evidence of statistical relationships was further assessed combining Venice Criteria and a false‐positive report probability test. Based on 20 publications with 15 969 cases and 17 174 controls, we found that six variants in miRNAs or biosynthesis genes that proved significant associations with LC risk, whereas five proved no association. Subgroup analyses by ethnicity and genetic models were performed, suggesting that four associations were rated as demonstrating strong evidence of relationship with LC risk, including *miRNA‐146a* rs2910164 in all populations under dominant model and in Asians under dominant and recessive models, and *AGO1* rs595961 in Asians under allelic model. Three associations were graded as moderate, and seven associations were rated as weak. This study presents the relationships between SNPs in miRNAs or biosynthesis genes and LC risk, subsequently demonstrates the credibility of these significant associations, and highlights the role in the pathogenesis of LC.

## Graphical abstract

Our study evaluated the cumulative evidence of significant associations between SNPs in miRNA or biosynthesis genes and lung cancer (LC) risk by combining Venice Criteria and false‐positive report probability test. Two variants with four associations were rated as demonstrating strong evidence with risk of LC, three associations were moderate, and seven associations were weak. Our paper provides summary evidence with relationships between SNPs in miRNAs or biosynthesis genes and LC risk, subsequently demonstrates the credibility of these significant associations, and highlights the role in the pathogenesis of LC.

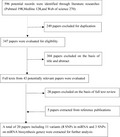

## 1 INTRODUCTION

Lung cancer (LC), a malignant carcinoma of respiratory system, remains the leading cause of cancer incidence and mortality.^1^ Worldwide, in 2018, estimated 2.1 million patients had LC, accounting for about 11.6% cancer incidence, followed by prostate and colorectal cancer, and estimated 1.8 million deaths, accounting for about 18.4% cancer deaths, followed by liver and stomach cancer.^1^ Studies showed that the development of LC attributes to the roles of both environmental exposure and the variations in gene^2^; more than 80% patients with LC have smoked, only less than 20% of smokers will eventually be diagnosed with LC; while nonsmokers with a family history of cancer have a higher LC risk, indicating that variations in gene play a key role in the pathogenesis of LC.^3^, ^4^ Many studies involving the relationships between single nucleotide polymorphisms (SNPs) and LC risk had been performed last decade and were taken consideration into as part of the genetic variant study, as well as the role of SNPs in microRNAs (miRNAs), which regulates up to 30% of gene‐related human disease.^5^, ^6^

MicroRNAs are a class of single‐stranded small noncoding RNAs of 19‐25 nucleotides in length that form base pairs with target messenger RNA to negatively regulate translation stability and efficiency via posttranscriptional gene silencing^7^; the study on its role in the pathogenesis of LC is also in further progress, offering a foundation for the future treatment of cancer via research on the specific mechanism of different SNPs in miRNA (miRNA‐SNPs).^8^, ^9^, ^10^ Moreover, abnormal expression of key genes and proteins in miRNA processing is an important reason for abnormal miRNA expression, thus increasing or decreasing tumor susceptibility.^9^, ^10^, ^11^, ^12^, ^13^, ^14^, ^15^ The miRNA biosynthesis genes, act as the key genes, including Argonaute proteins (*AGO1*, *AGO2*, and *HIWI*), human immunodeficiency virus transactivating response RNA binding protein, *GEMIN3*, and *GEMIN4*, formed miRNA‐induced silencing complex (*RISC*), which incorporate one strand of the miRNA duplex during the synthesis process of miRNAs, play a role by inhibiting the expression of target genes and hence influence the genesis and development of human cancer.^16^, ^17^, ^18^, ^19^ The research on miRNA biosynthesis genes has been gradually started and deepened in recent years, with more and more comprehensive and in‐depth results gradually appeared. And previous studies have shown that miRNA biosynthesis genes are closely related to the risk of LC, especially *AGO1* and *GEMIN4*.^20^

As early as 2002, Calin et al first found that miRNA expression has been linked with malignant tumors in human, and discovered that the miRNA (*miRNA*‐15a and *miRNA‐16‐1*) expression with down‐regulation or deletion has been linked with B‐cell chronic lymphoblastic leukemia.^11^ In 2005, a study conducted by Johnson et al presented that *miRNA let‐7* had a negative correlation with *RAS* protein expression in human LC cell line^12^; the similar report also showed that *let‐7* expression in microarray analysis of human cancer tissues was decreased in LC tissues, rather than in adjacent normal lung tissues.^13^ Additional studies also reported that miRNA have been associated with LC risk, including the up‐regulation of *miRNA‐17‐92* cluster in LC.^14^ Subsequently, some studies also reported that the SNPs in miRNAs have been linked with the pathogenesis of LC.^9^ As early as 2008, Jazdzewski et al first reported that *miR‐146a* rs2910164 triggered the down‐regulation of mature *miR‐146a*, which could affect the targeted binding of mRNA, and then regulate the development of LC.^15^ Then, studies based on miRNA‐SNPs have gradually appeared in the public. To date, more than 36 miRNA‐SNPs have been taken consideration into as part of research such as *miR‐146a* rs2910164, *miR‐143/145* rs4705343, *miR‐196a2* rs11614913, *miR‐499* rs3746444, *miR‐608* rs4919510, *miR‐27a* rs895819, *miR‐149* rs2292832, *miR‐219‐1* rs213210 and so on.

Although several studies based on SNPs in miRNAs and risk of LC have been performed, the results for same SNP in miRNAs in different studies have been disputed and are controversial, indicating the possibility of false‐positive associations. Therefore, we carried out meta‐analyses to further evaluate the credibility of these relationships between SNPs in miRNAs or biosynthesis genes and LC risk.

## 2 METHODS

All methods were in accordance with the guidelines of the Preferred Reporting Items for Systematic Reviews and Meta‐Analyses Statement, the Human Genome Epidemiology Network for systematic review of genetic association studies and Meta‐analysis of Observational Studies in Epidemiology guidelines.^21^, ^22^, ^23^, ^24^, ^25^

### 2.1 Literature search

PubMed, Medline, and Web of Science were used to search for relevant articles published before 30 May 2019, by using the following terms: (“lung”) and (“tumor” or “malignant” or “malignancy” or “neoplasm” or “neoplasia” or “oncology” or “cancer” or “carcinoma” or “adenocarcinoma”) and (“variant” or “variation” or “genotype” or “polymorphism” or “single nucleotide polymorphism” or “SNP”) and (“MicroRNA” or “miRNA” or “MiRNA”). In addition, the references in included articles were also checked to obtain other potential relevant data.

### 2.2 Criteria for inclusion and exclusion

The following criteria should be considered with selection of studies: (a) investigating relationships between miRNA‐SNPs or biosynthesis genes and risk of LC with studies performed in a case‐control or cohort design in human; (b) patients with LC were pathologically or histologically confirmed; (c) presenting the sample size in cases and controls; wherever necessary, the amount of genotype and/or allelic distributions should be offered; (d) the published or online full text in the journals was in English. Studies were excluded if: (a) studies had insufficient relevant data; (b) studies were published by form of conference abstracts and letters to editors, rather than as full reports; (c) the studies were mainly based on LC survival/mortality rate (rather than incidence).

### 2.3 Data extraction

The relevant information was independently extracted by two authors (GL and JT) and were crosschecked each other. Any disagreement was discussed with the third investigator (HC) and finally resolved together. For the qualified SNPs, the following publication details were extracted, including first author, the year of publishing, study design, ethnicity, gene name, variation in gene, the sample size in cases and controls, minor allelic frequency (MAF), genotype counts for cases and controls. Specifically, two major ethnicities, Asian and Caucasian, were frequently reported in our study; “all populations” indicate two or more. If the same study population was reported in more than one article, we collected data from the most recently published study with the greatest number of and most integrated participants. As for the same genetic variant, the modes of presentation were inconsistent, we therefore first checked on the website (https://www.ncbi.nlm.nih.gov/snp/) and then used the most recent one. We used the Newcastle Ottawa Scale (NOS) to evaluate the quality of included eligible studies.^26^ A maximum of nine scores was assigned to each article: four scores for the assessment of selection, two scores for comparability, and three scores for outcomes. The ranges of NOS score were divided into three grades: 0‐3 (low quality), 4‐6 (moderate quality), 7‐9 (high quality).

### 2.4 Statistical analysis

We carried out comprehensive meta‐analyses using allelic, dominant, and recessive models (See Table S3); wherever necessary, a subgroup analysis based on ethnicity was also investigated. Cochran's *Q* test and the *I* ^2^ statistic were used to evaluate the heterogeneity among different publications.^27^, ^28^ Briefly, the values of *I* ^2^ were divided into three grades: ≤25%, 25%‐50%, ≥50% (indicating no or little heterogeneity, moderate heterogeneity, and large heterogeneity, respectively). Additionally, we adopted the random effect model if the *P*‐value was <.1, otherwise the fixed effect model was used. Moreover, we performed sensitivity analyses for all SNPs, especially for SNPs with significant associations, further to assess whether the significant ORs were robust by excluding a single study (dataset), or the first published study, or studies deviated from the Hardy‐Weinberg equilibrium (HWE) in the controls. Begg's test and Egger's test were used to evaluate potential publication bias and small study bias, respectively.^29^, ^30^ Probability of an excess of significant findings for an individual meta‐analysis was also performed.^31^ A *P*‐value <.05 in the meta‐analysis and <.1 in the Cochran's *Q*, Begg's and Egger's tests, as well as a test for excess significant findings, was considered significant, as recommended.^29^, ^30^, ^31^ Statistical analyses were conducted using Stata, version 12 (Stata).

### 2.5 Evaluation of cumulative evidence

First, the Venice Criteria were used to evaluate the epidemiological credibility of significant associations identified by the meta‐analyses.^21^ The strength of cumulative evidence was rated as strong, moderate, or weak based on criteria in addition to amount of evidence, replication of association, and protection from bias (grades of A, B or C were assigned based on each criterion noted above). The amount of evidence was evaluated by totaling the number of alleles or genotypes among the cases and controls; it was then segmented into three levels: >1000, 100‐1000, and <100, indicating grades A, B, and C, respectively. Heterogeneity statistics was used to assess the replication of association and its range was divided into three levels: grade A (*I* ^2^ ≤ 25%), grade B (25% < *I* ^2^ < 50%), or grade C (*I* ^2^ ≥ 50%). Protection from bias was mainly determined using sensitivity analysis and a series of bias tests including publication bias and small‐study bias, as well as an excess of significant findings. Generally, grade A was assigned to indicate no observable bias, or if bias was unlikely to explain the presence of the association, grade B was assigned if bias could be present, and grade C was allocated if the bias was evident or was likely to explain the presence of the association. We utilized an extensive checklist to check the sources of bias in different settings proposed by the Venice Criteria (see Supporting Information notes). The magnitude of the association was related to the evaluation of protection from bias; a summary OR < 1.15 (or >0.87 in a protection effect) was graded as a score of C for an association, unless the association had been replicated, prospectively, by several studies with no evidence of publication bias (ie, GWAS or GWAS meta‐analysis from collaborative studies).^23^ Cumulative epidemiological evidence of significant associations was then assigned one of three groups: strong association (A was assigned to all three grades), weak association (C was assigned to any of the grades), or moderate association (a combination of A, B, and C).

As Wacholder et al suggested, a prior probability of 0.05 and a false‐positive report probability (FPRP) cutoff value of 0.2 in FPRP assay should be performed to detect the potential false‐positive results among significant associations and assess whether these associations should be omitted.^32^ Statistical power and FPRP values were calculated using the Excel spreadsheet obtained on Wacholder's website.^32^ If the calculated FPRP value was below the prespecified noteworthiness value of 0.2, we considered the association noteworthy, indicating the association might be true. Three levels were assigned based on the FPRP value: strong (FPRP < 0.05), moderate (0.05 ≤ FPRP ≤0.2), or weak (FPRP >0.2). An FPRP < 0.05 triggered an upgrade of cumulative evidence from moderate to strong or from weak to moderate. Otherwise, an FPRP >0.2 triggered a downgrade of cumulative evidence from strong to moderate or from moderate to weak.

## 3 RESULTS

### 3.1 Characteristics of eligible studies

**Figure 1:**
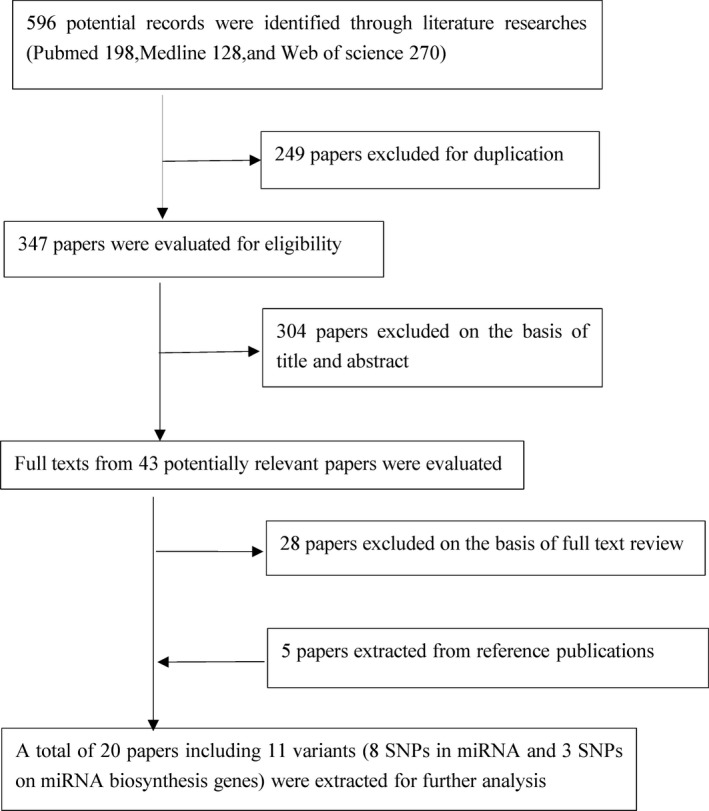
Flow diagram of search strategy and study selection. SNP, single nucleotide polymorphism

Our search yielded a total of 596 publications. As presented Figure 1, 249 papers were excluded for duplication, 304 were excluded on the basis of title and abstract, and 28 were excluded after a full text review. Moreover, five studies were screened from the reference publication. Ultimately, a total of 20 articles with 15 969 cases and 17 174 controls were eligible to assess the relationships between 11 SNPs and LC risk (eight SNPs in miRNAs and three SNPs on biosynthesis genes) after ruling out SNPs which appear in only one article (For example, Li D et al analyzed 11 SNPs, data of eight SNPs were not extracted because of absence in any other articles). The characteristics of the included articles are presented in Table 1. The sample size ranged from 230 to 7733, and the publication years ranged from 2009 to 2018; 16 papers (80%) were published over the past 5 years. The participants presented in all the eligible articles were performed in a case‐control study. Additionally, the mean score of study quality for the included papers was 6.85, and the scores for all of the eligible studies were >5 (see Table S2).

**Table 1:** Characteristics of the included articles

Study, y^a^	Study design	Country/region	Ethnicity	Dataset^b^	Variant	Gene^c^/miRNA	Case	Control
Liu Z (2018)	CCS	China	Asian	4	rs2910164 (C>G)	miR‐146a	1024	1058
					rs11614913 (C>T)	miR‐196a2	1006	1051
					rs7372209 (T>C)	miR‐26‐1	1010	1062
					rs895819 (T>C)	miR‐27a	1006	1026
Yin Z (2017)	CCS	China	Asian	1	rs11614913 (C>T)	miR‐196a2	1003	1003
Fan L (2017)	CCS	China	Asian	1	rs12220909 (G>C)	miR‐4293	995	1454
Yin Z (2017)	CCS	China	Asian	1	rs2910164 (C>G)	miR‐146a	1131	1003
Li H (2016)	CCS	China	Asian	1	rs2292832 (T>C)	miR‐149	555	395
Fang X (2016)	CCS	China	Asian	3	rs7813 (C>T)	GEMIN4^c^	473	395
					rs910924 (C>T)	GEMIN4^c^	473	395
					rs595961 (A>G)	AGO1^c^	473	395
Li D (2016)	CCS	China	Asian	3	rs3746444 (A>G)	miR‐499	1200	1200
					rs4919510 (C>G)	miR‐608	1200	1200
					rs12220909 (G>C)	miR‐4293	500	500
Yin Z (2016)	CCS	China	Asian	3	rs2910164 (C>G)	miR‐146a	575	608
					rs4919510 (C>G)	miR‐608	575	608
					rs895819 (T>C)	miR‐27a	575	608
Yin Z (2016)	CCS	China	Asian	1	rs7372209 (T>C)	miR‐26‐1	268	266
Sodhi KK (2015)	CCS	Others	Asian	2	rs2910164 (C>G)	miR‐146a	250	255
					rs11614913 (C>T)	miR‐196a2	250	255
Ma JY (2015)	CCS	China	Asian	1	rs895819 (T>C)	miR‐27a	542	557
Jia Y (2014)	CCS	China	Asian	1	rs2910164 (C>G)	miR‐146a	400	400
Jeon HS (2014)	CCS	Korea	Asian	1	rs2910164 (C>G)	miR‐146a	1091	1096
Vinci S (2012)	CCS	Italy	Caucasian	4	rs2910164 (C>G)	miR‐146a	101	129
					rs2292832 (T>C)	miR‐149	101	129
					rs11614913 (C>T)	miR‐196a2	101	129
					rs3746444 (A>G)	miR‐499	101	129
Hong YS (2011)	CCS	Korea	Asian	1	rs11614913 (C>T)	miR‐196a2	406	428
Kim JS (2010)	CCS	Korea	Asian	3	rs595961 (A>G)	AGO1^c^	98	97
					rs910924 (C>T)	GEMIN4^c^	93	90
					rs7813 (C>T)	GEMIN4^c^	98	99
Tian T (2009)	CCS	China	Asian	4	rs2910164 (C>G)	miR‐146a	1058	1035
					rs2292832 (T>C)	miR‐149	1058	1035
					rs11614913 (C>T)	miR‐196a2	1058	1035
					rs3746444 (A>G)	miR‐499	1058	1035
Yin Z (2015)	CCS	China	Asian	3	rs11614913 (C>T)	miR‐196a2	258	310
					rs2910164 (C>G)	miR‐146a	258	310
					rs4919510 (C>G)	miR‐608	258	310
Kim MJ (2010)	CCS	Korea	Asian	1	rs11614913 (C>T)	miR‐196a2	654	640
Xie K (2017)	CCS	China	Asian	1	rs12740674 (C>T)	miR‐1262	3387	4346

Abbreviation: CCS, case‐control study.

a References for the 20 included articles are presented in the Supporting Information.

b Datasets represented the number of datasets in the original publications.

c MicroRNA (miRNA) biosynthesis genes or a variant of gene located in a miRNA binding site.

### 3.2 Main meta‐analyses

We conducted meta‐analyses to assess the relationships between the miRNA‐SNPs or biosynthesis genes and LC risk. These results are presented in Table 2. Six variants demonstrated a nominally significant association with LC risk, including three SNPs in miRNAs (*miRNA‐146a* rs2910164, *miRNA‐499* rs3746444, and *miRNA‐27a* rs895819) and three SNPs on miRNA‐related biosynthesis genes (*AGO1* rs595961, *GEMIN4* rs7813, and *GEMIN4* rs910924). Specifically, our study demonstrated a significant association between *miRNA‐146a* rs2910164 and LC risk in all populations (allelic model: OR = 0.886, 95% CI = 0.841‐0.934, *P* < .001; dominant model: OR = 0.835, 95% CI = 0.769‐0.906, *P* < .001; recessive model: OR = 0.875, 95% CI = 0.801‐0.956, *P* = .003), and in Asians (allelic model: OR = 0.889, 95% CI = 0.843‐0.937, *P* < .001; dominant model: OR = 0.834, 95% CI = 0.768‐0.905, *P* < .001; recessive model: OR = 0.885, 95% CI = 0.809‐0.968, *P* = .008), but demonstrated no significant association in Caucasians under the three genetic models. For *miRNA‐499* rs3746444, we found that SNP rs3746444 under dominant model had significant association with LC risk in all populations (OR = 1.142, 95% CI = 1.009‐1.294, *P* = .036), and a nominally significant association between SNP rs3746444 and risk of LC was found in Asians under recessive model (OR = 1.403, 95% CI = 1.034‐1.904, *P* = .029), rather than the allelic or the dominant model, as well as in Caucasians under all three models.

**Table 2:** Associations between variants in the microRNAs/biosynthesis gene with risk of lung cancer

Gene	Variant	Allelics	Ethnicity	Number evaluation		MAF^a^	Genetic models	Effect model	Risk of Meta‐analysis		Heterogeneity		Venice Criteria^b^	FPRP^c^	Credibility of evidence
				Studies	Cases/controls				OR (95% CI)	*P*	*I* ^2^ (%)	*P*_Q_			
miR‐146a	rs2910164	**G**/C	All	9	5888/5894	**0.49**	**Allelic**	Fixed	**0.886 (0.841‐0.934)**	<.001	34.4	0.143	ABC	**<0.001**	**Moderate**
							**Dominant**	Fixed	**0.835 (0.769‐0.906)**	<.001	0	0.512	AAA	**<0.001**	**Strong**
							**Recessive**	Fixed	**0.875 (0.801‐0.956)**	.003	25.2	0.219	ABC	0.124	**Weak**
			Asian	8	5787/5765	**0.49**	**Allelic**	Fixed	**0.889 (0.843‐0.937)**	<.001	37.6	0.13	ABC	**<0.001**	**Moderate**
							**Dominant**	Fixed	**0.834 (0.768‐0.905)**	<.001	2.1	0.414	AAA	**<0.001**	**Strong**
							**Recessive**	Fixed	**0.885 (0.809‐0.968)**	.008	17.8	0.29	AAA	0.126	**Strong**
			Caucasian	1	101/129	**0.74**	Allelic	Fixed	0.723 (0.482‐1.084)	NA	NA	NA			
							Dominant	Fixed	0.953 (0.379‐2.396)	NA	NA	NA			
							Recessive	Fixed	0.592 (0.350‐1.001)	NA	NA	NA			
miR‐196a2	rs11614913	**T**/C	All	8	4736/4851	0.53	Allelic	Random	0.952 (0.848‐1.070)	NA	NA	NA			
							Dominant	Random	0.985 (0.835‐1.162)	.854	62.3	0.01			
							Recessive	Random	0.878 (0.710‐1.087)	.233	76.3	0			
			Asian	7	4635/4722	0.53	Allelic	Random	0.928 (0.829‐1.039)	.196	71.4	0.002			
							Dominant	Random	0.950 (0.809‐1.115)	.528	60	0.02			
							Recessive	Random	0.853 (0.688‐1.059)	.149	78.2	0			
			Caucasian	1	101/129	0.31	Allelic	Random	1.375 (0.934‐2.023)	NA	NA	NA			
							Dominant	Random	1.540 (0.900‐2.635)	NA	NA	NA			
							Recessive	Random	1.604 (0.664‐3.880)	NA	NA	NA			
miR‐4293	rs12220909	**C**/G	Asian	2	1795/1954	0.20	Allelic	Random	0.853 (0.659‐1.104)	.227	75.3	0.044			
							Dominant	Random	0.799 (0.611‐1.046)	.102	67.4	0.08			
							Recessive	Fixed	1.031 (0.724‐1.466)	.866	0	0.475			
miR‐149	rs2292832	**C**/T	All	3	1714/1559	0.36	Allelic	Fixed	0.996 (0.898‐1.104)	.935	45.8	0.158			
							Dominant	Fixed	1.012 (0.879‐1.165)	.87	42.4	0.176			
							Recessive	Fixed	0.957 (0.775‐1.181)	.681	4.3	0.352			
			Asian	2	1613/1430	0.33	Allelic	Fixed	1.020 (0.916‐1.135)	.723	0	0.321			
							Dominant	Fixed	1.035 (0.897‐1.195)	.638	0	0.507			
							Recessive	Fixed	1.001 (0.795‐1.260)	.996	17	0.272			
			Caucasian	1	101/129	0.71	Allelic	Fixed	0.724 (0.489‐1.073)	NA	NA	NA			
							Dominant	Fixed	0.495 (0.219‐1.121)	NA	NA	NA			
							Recessive	Fixed	0.760 (0.450‐1.283)	NA	NA	NA			
miR‐499	rs3746444	**G**/A	All	4	2359/2364	0.16	Allelic	Random	1.157 (0.951‐1.407)	.145	65.9	0.032			
							Dominant	Fixed	1.142 (1.009‐1.294)	.036	51.1	0.105	ACC	0.415	Weak
							Recessive	Fixed	1.335 (0.999‐1.785)	.051	34.8	0.203			
			Asian	3	2258/2235	0.16	Allelic	Random	1.186 (0.942‐1.493)	.146	76.1	0.015			
							Dominant	Random	1.176 (0.936‐1.477)	.164	67.1	0.048			
							**Recessive**	Fixed	**1.403 (1.034‐1.904)**	.029	42.4	0.176	BBC	0.459	**Weak**
			Caucasian	1	101/129	0.27	Allelic	Random	1.005 (0.664‐1.520)	NA	NA	NA			
							Dominant	Random	1.075 (0.638‐1.811)	NA	NA	NA			
							Recessive	Fixed	0.799 (0.298‐2.140)	NA	NA	NA			
miR‐608	rs4919510	**G**/C	Asian	4	2033/2118	0.44	Allelic	Random	1.063 (0.849‐1.331)	.596	84.2	0			
							Dominant	Random	1.128 (0.896‐1.419)	.305	63.0	0.044			
							Recessive	Random	1.047 (0.738‐1.484)	.797	80.6	0.001			
AGO1^d^	rs595961	**G**/A	Asian	2	571/492	0.85	**Allelic**	Fixed	**0.692 (0.551‐0.869)**	.022	0	0.852	AAA	**0.044**	**Strong**
							Dominant	Fixed	0.558 (0.245‐1.273)	.166	35.9	0.212			
							**Recessive**	Fixed	**0.656 (0.506‐0.851)**	.002	0	0.482	BAA	0.059	**Moderate**
miR‐26‐1	rs7372209	**C**/T	Asian	2	1278/1328	0.70	Allelic	Fixed	0.964 (0.857‐1.085)	.548	0	0.592			
							Dominant	Fixed	0.937 (0.716‐1.226)	.633	45	0.177			
							Recessive	Fixed	0.961 (0.824‐1.121)	.611	43.3	0.184			
GEMIN4^d^	rs7813	**T**/C	Asian	2	571/494	0.64	**Allelic**	Fixed	**1.258 (1.050‐1.507)**	.013	62.2	0.104	ACA	0.199	**Weak**
							Dominant	Fixed	1.097 (0.756‐1.593)	.625	0	0.988			
							Recessive	Random	1.227 (0.627‐2.403)	.55	78.8	0.03			
miR‐27a	rs895819	**C**/T	Asian	3	2123/2191	0.26	Allelic	Random	1.067 (0.903‐1.261)	.446	66.4	0.051			
							Dominant	Fixed	1.024 (0.908‐1.154)	.705	37.8	0.2			
							**Recessive**	Fixed	**1.292 (1.041‐1.602)**	.02	48	0.146	BBC	0.289	**Weak**
GEMIN4^d^	rs910924	**T**/C	Asian	2	566/485	0.15	**Allelic**	Fixed	**0.732 (0.570‐0.940)**	.015	0	0.35	BAA	0.264	**Weak**
							**Dominant**	Fixed	**0.704 (0.532‐0.931)**	.014	0	0.336	BAA	0.288	**Weak**
							Recessive	Fixed	0.689 (0.283‐1.676)	.411	0	0.801			

Abbreviations: A, adenine; C, cytosine; G, guanine; T, thymine; OR, odds ratio; CI, confidence interval; MAF, minor allelic frequency in control; NA, not applicable; FPRP, false‐positive report probability.

a Allelics: Minor allelic (bold) vs major allelic.

b Venice criteria grades are for amount of evidence, replication of the association and protection from bias.

c The prior probability of FPRP is 0.05, and the FPRP level of noteworthiness is 0.20.

d MicroRNA (miRNA) biosynthesis genes.

The rest of four SNPs significantly associated with LC risk were exclusively performed in Asians; significant associations were observed in *AGO1* rs595961 under the allelic and the recessive models (allelic model: OR = 0.692, 95% CI = 0.551‐0.869, *P* = .022; recessive model: OR = 0.656, 95% CI = 0.506‐0.851, *P* = .002, respectively), rather than the dominant model. For *miRNA‐27a* rs895819, our study presented that SNP rs895819 had a nominally significant association with LC risk under the recessive model (OR = 1.292, 95% CI = 1.041‐1.602, *P* = .02), but not the allelic or the dominant model. For *GEMIN4*, two SNPs (rs7813 and rs910924) had significant association with LC risk; the former SNP had significant association with an increased risk of LC under the allelic model (OR = 1.258, 95% CI = 1.051‐1.507, *P* = .013), rather than the dominant or the recessive model; the latter variant had significant association with an decreased risk of LC under the allelic and the dominant models (allelic model: OR = 0.732, 95% CI = 0.570‐0.940, *P* = .015, dominant model: OR = 0.704, 95% CI = 0.532‐0.931, *P* = .014), rather than the recessive model.

In addition, current study found that five variants in five miRNAs had no significant association with LC risk under all three genetic models, including *miRNA‐196a2* rs11614913, *miRNA‐4293* rs12220909, *miRNA‐149* rs2292832, *miRNA‐608* rs4919510, and *miRNA‐26‐1* rs7372209; of these five variants, two variants were performed both in Asians and Caucasians and three variants concentrated on single (Asian) population.

### 3.3 Cumulative evidence of association

Cumulative epidemiological evidence was graded for six SNPs associated with LC risk; the details of the evidence are presented in Table 2. We first assessed these associations using Venice Criteria. In terms of the amount of evidence, nine grade A, five grade B, and zero grade C were assigned to further evaluate the credibility of evidence. With regards to replication of association, seven grade A, five grade B and two grade C were assigned for further evaluation. Based on protection from bias, eight grade A, zero grade B and six grade C were assigned for further assessment. Evidence for relationship with LC risk was thereby rated as strong for four associations (*miRNA‐146a* rs2910164 in all populations under dominant model and in Asians under the dominant and the recessive models; *AGO1* rs595961 in Asians under the allelic model), moderate for three associations (*AGO1* rs595961 in Asians under the recessive model; *GEMIN4* rs910924 in Asians under the allelic and the dominant model), and weak for seven associations (*miRNA‐146a* rs2910164 in all populations under the allelic and the recessive models, and in Asians under the allelic model; *miRNA‐499* rs3746444 in all populations under the dominant model, and in Asians under the recessive model; *GEMIN4* rs7813 in Asians under the allelic model; *miR‐27a* rs895819 in Asian under the recessive model) based on Venice Criteria.

**Figure 2:**
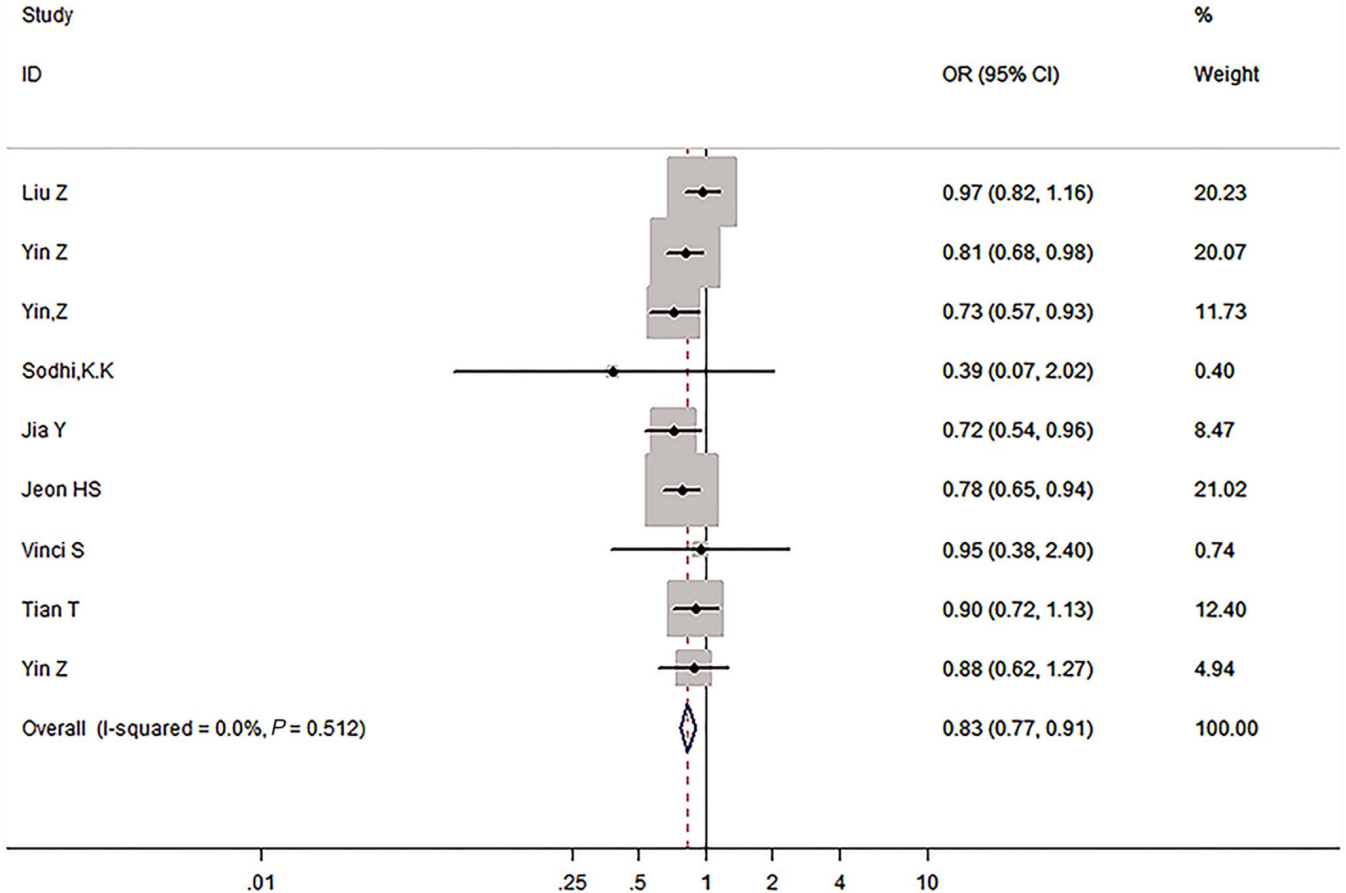
The forest plot of strong cumulative evidence association between miR‐146a rs2910164 and lung cancer risk in all population under the dominant model

**Figure 3:**
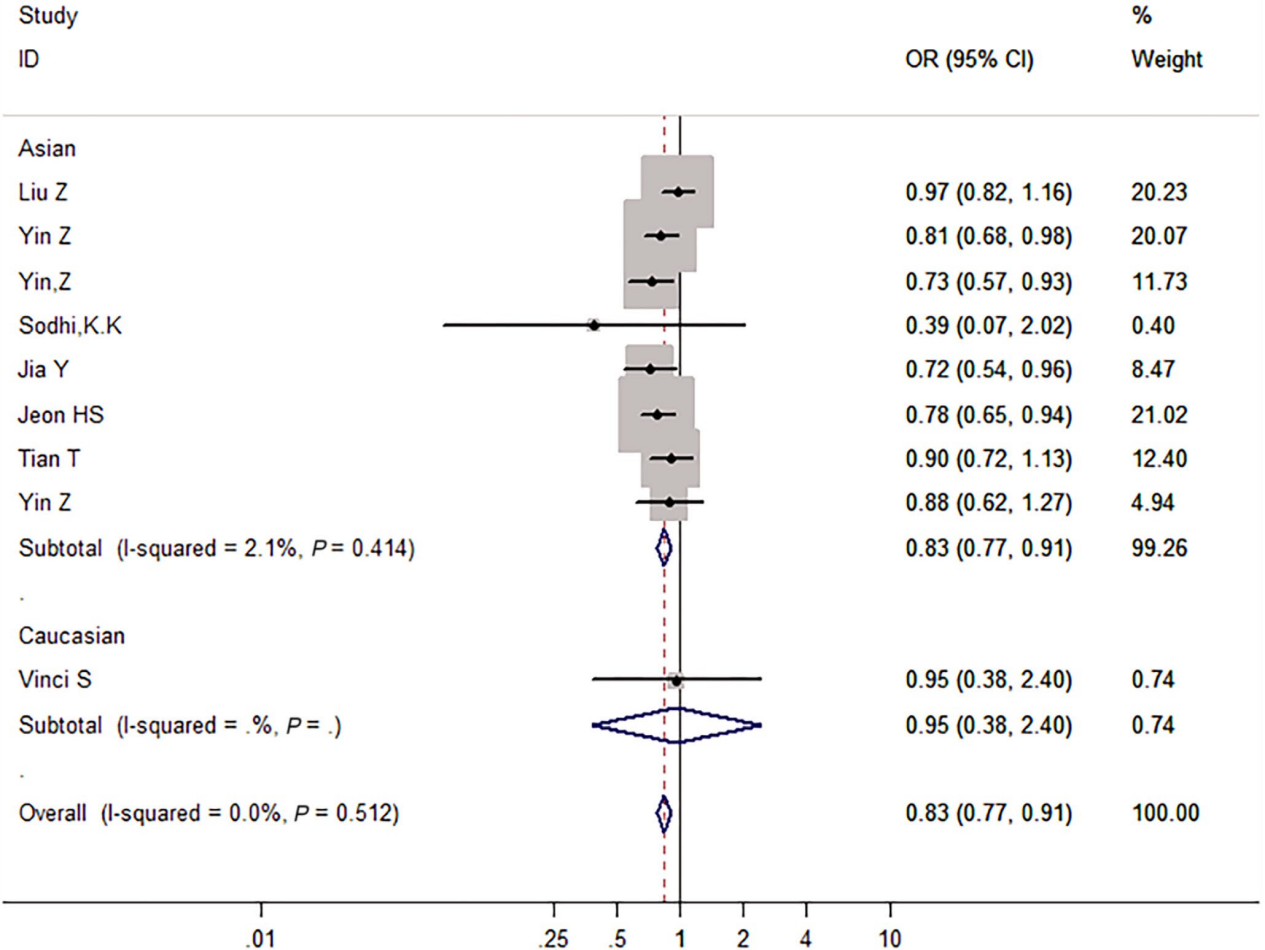
The forest plot of strong cumulative evidence association between miR‐146a rs2910164 and lung cancer risk in Asian under the dominant model

**Figure 4:**
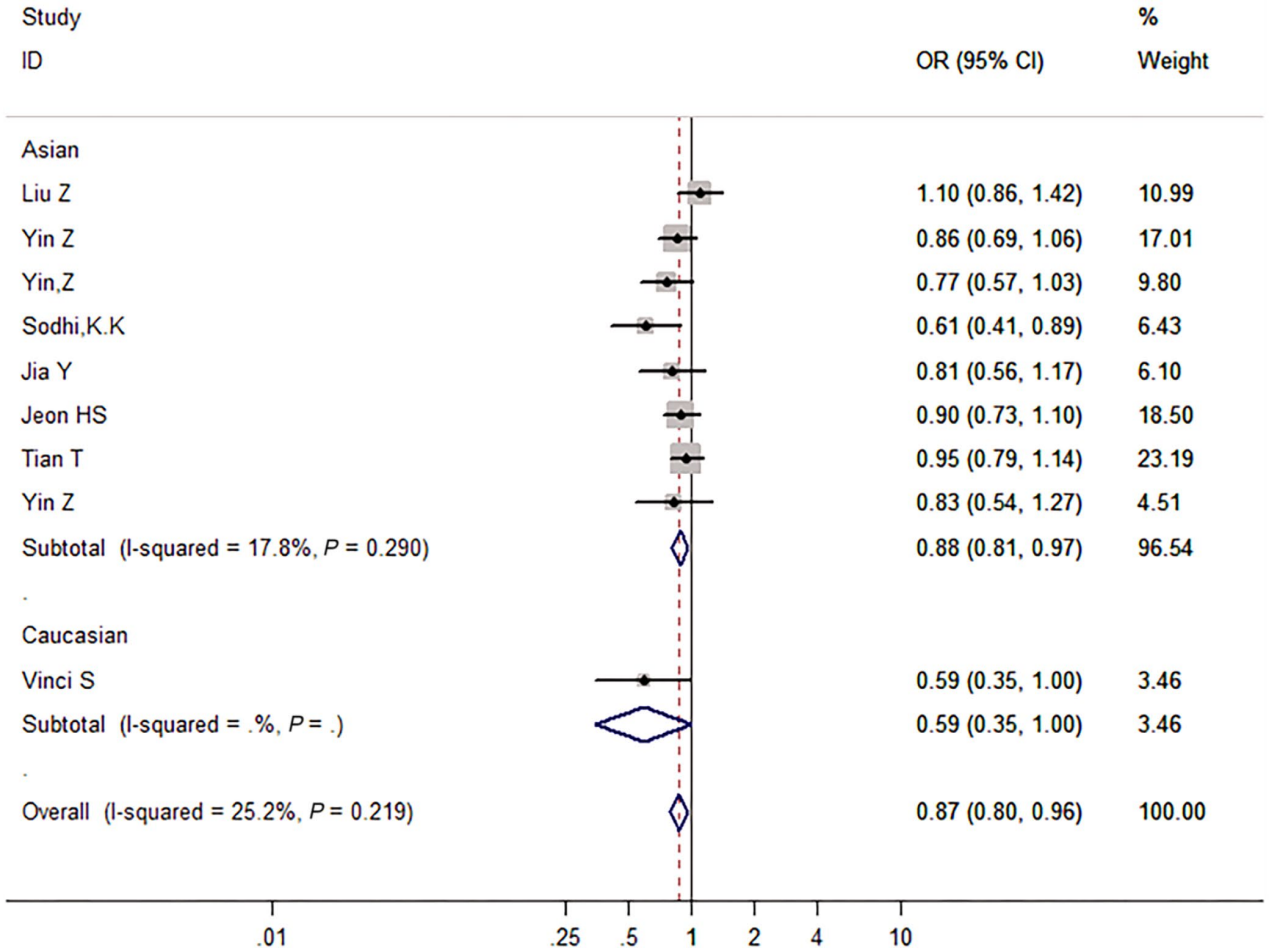
The forest plot of strong cumulative evidence association between miR‐146a rs2910164 and lung cancer risk in Asian under the recessive model

**Figure 5:**
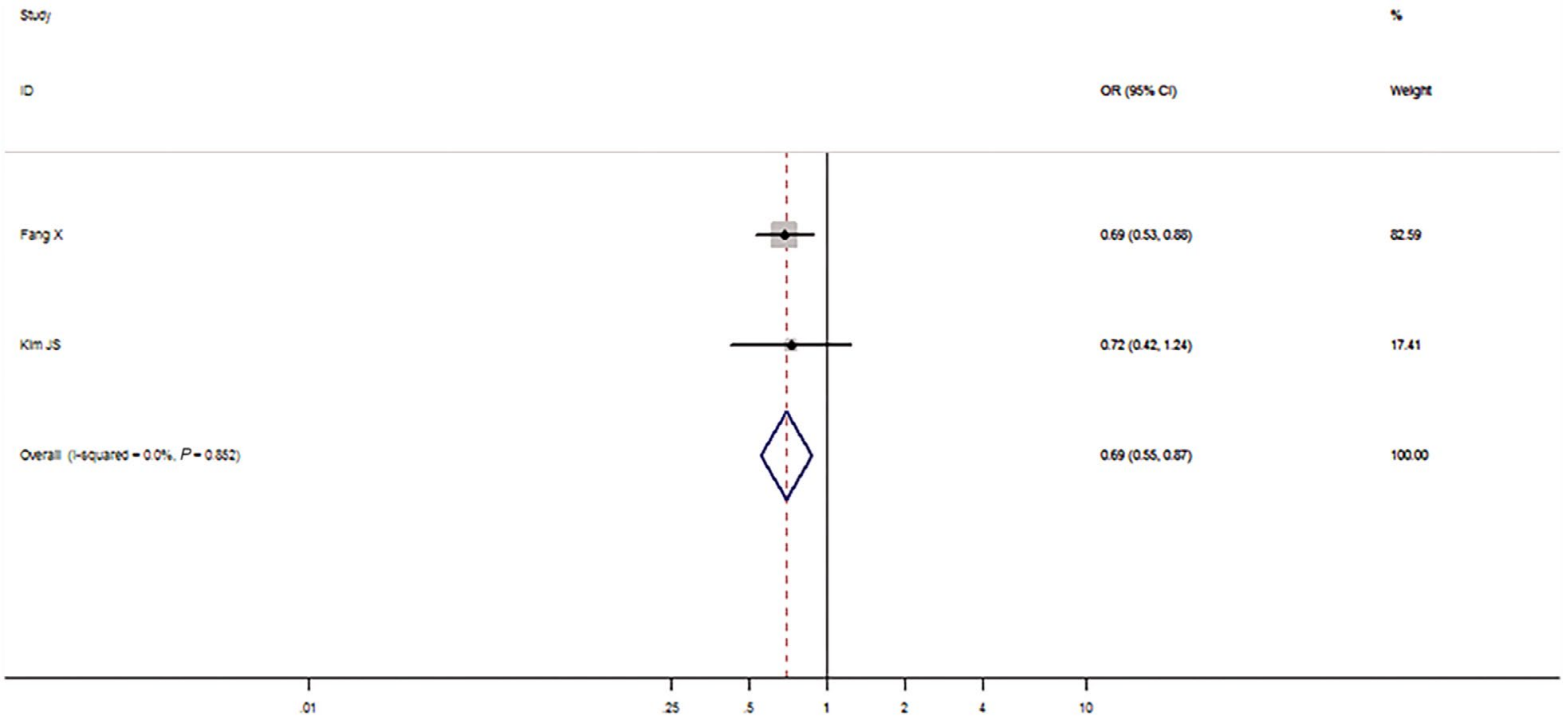
The forest plot of strong cumulative evidence association between AGO1 rs595961 and lung cancer risk in Asian population under the allelic model

We then assessed the probability of a true association with LC risk for the nominally significant variants through calculating their FPRP values. Relationships with LC risk presented an *P*‐value of FPRP assay less than .05 for two associations (*miRNA‐146a* rs2910164 in all and Asian populations under the allelic model), from .05 to .2 for nine associations, and greater than .2 for the rest of three associations (*miRNA‐499* rs3746444 in all populations under the dominant model, *GEMIN4* rs910924 in Asians under the allelic and the dominant models). Therefore, cumulative epidemiological evidence of an association was rated as strong for *miRNA‐146a* rs2910164 in all populations under the dominant model and in Asians under the dominant and the recessive models, and for AGO1 rs595961 in Asians under the allelic model (see Figures 2, 3, 4, 5); moderate for *miRNA‐164a* rs2910164 in all and Asian populations under the allelic model, and for *AGO1* rs595961 in Asians under the recessive model; weak for *miRNA‐146a* rs2910164 in all populations under the recessive model, for *miRNA‐499* rs3746444 in all populations under the dominant model, and for other associations in Asians, including *miRNA‐499* rs3746444 and *miRNA‐27a* rs895819 under the recessive model, *GEMIN4* rs7813 under the allelic model, and *GEMIN4* rs919024 under the allelic and the dominant models.

### 3.4 Heterogeneity, bias, and sensitivity analyses

The evaluations of heterogeneity, bias, and sensitivity analyses in our study are presented in Table 2, low heterogeneity was found for relationships of rs2910164 (allelic model: *I* ^2^ = 0.0%, *P* = .512) in all populations, rs2910164 (dominant model: *I* ^2^ = 2.1%, *P* = .414, recessive model: *I* ^2^ = 17.8%, *P* = .29, respectively) in Asians, rs595961 (allelic model: *I* ^2^ = 0.0%, *P* = .852, recessive model: *I* ^2^ = 0.0%, *P* = .482, respectively) in Asians, and rs910924 (allelic model: *I* ^2^ = 0.0%, *P* = .35, dominant model: *I* ^2^ = 0.0%, *P* = .336, respectively) in Asians; moderate heterogeneity was detected for relationships of rs2910164 (allelic model: *I* ^2^ = 34.4%, *P* = .143, recessive model: *I* ^2^ = 25.2%, *P* = .219, respectively) in all populations, rs2910164 (allelic model: *I* ^2^ = 37.6%, *P* = .13) in Asians, rs3746444 (recessive model: *I* ^2^ = 42.4%, *P* = .176) in Asians, and rs895819 (recessive model: *I* ^2^ = 48%, *P* = .146) in Asians; large heterogeneity was found for relationship of rs3746444 (dominant model: *I* ^2^ = 55.1%, *P* = .105) in all population, and rs7813 (allelic model: *I* ^2^ = 62.2%, *P* = .104) in Asians. There was little evidence of publication bias for associations of SNPs with LC risk (*P* > .10 for all tests), except for rs2910164 in all populations under the allelic and the recessive models, and in Asians under the allelic model (*P* < .10). In addition, we performed sensitivity analyses to assess the robustness of results of these significant relationships and found that removal of a single study (dataset), the first published or studies deviated from HWE in controls did not change the summary ORs (data not shown), except for the rs895819 in Asians under the recessive model and the rs3746444 in all populations under the dominant model and in Asians under the recessive model. In our sensitivity analyses, after the exclusion of study deviated from HWE in controls, no significant association was observed in the three genetic models above. In addition, three significant associations did not remove a single study (dataset) due to only two primary datasets, including rs595961, rs7813, rs910924 in Asians under allelic model and rs910924 in Asians under the dominant model (see Figures S7.1, S9.3, S11.3, and S11.6). The excess of significant findings was not assessed because genotype amounts from most studies were unavailable.

## 4 DISCUSSION

To our knowledge, our paper is the largest and most comprehensive assessment of literatures on the relationships between miRNA in SNPs or biosynthesis genes and LC risk done thus far. Although a large number of studies have reported the relationships between miRNAs‐SNPs or biosynthesis genes and LC risk, these results are inconsistent and controversial. Therefore, we extracted useful data from published papers to conduct meta‐analyses based on 20 articles including with 15 969 cases and 17 174 controls. We then assessed the credibility of this cumulative epidemiological evidence of nominally significant associations combining Venice Criteria and FPRP tests. Finally, four associations were graded as demonstrating strong evidence of relationship with LC risk, including *miRNA‐146a* rs2910164 in all population under the dominant model and in Asians under the dominant and the recessive models, and *AGO1* rs595961 in Asians under the allelic model. Three associations were graded as proving moderate evidence of relationship with risk of LC, and seven associations were rated as weak evidence.

*MiRNA‐146a*, located in 3p strand, may trigger a change for *MiRNA‐146a* from C to G in stem structure and control its expression and then promote the development of cancer susceptibility.^33^, ^34^ Our study provides strong evidence for relationships between SNP rs2910164 and risk of LC via a dominant model, with a 1.165‐fold decreased risk of LC in all population with a total sample size of 11 782. In the stratified analysis by ethnicity, our paper showed that SNP rs2910164 could decrease the risk of LC via under the dominant and the recessive models in Asians (with a total sample size of 11 552), rather than in Caucasians (with a total sample size of 230). However, the sample size in Caucasians was not fruitful; a large number of studies on this polymorphism to Caucasians are necessary in the future.

*AGO1*, located at chromosome 1p34‐35, is frequently deleted in human cancers, including LC.^35^, ^36^ Some studies have suggested that *AGO1* is a class of miRNA‐related biosynthesis genes, involving the negative regulation of the translation and stability of target mRNA; this gene was an important component of the *RISC* complex with *AGO2* and *DICER* and also plays a crucial role in miRNA‐mediated gene regulation^37^, ^38^, ^39^ and participates in the development of LC. Our study showed that there was strong evidence for a relationship between SNP rs595961 and risk of LC with a sample of 1063 Asians; the mutant G allele could decreased the risk of LC compared with the wild‐type A allele (OR = 0.692, 95% CI = 0.551‐0.869).^40^ However, this study sample was limited to single ethnic group (Asian), and involved a large proportion of Chinese participants. Further studies on this SNP and investigations into other ethnicities are recommended.

Three associations were graded as demonstrating moderate evidence of relationship with risk of LC, including *miRNAs‐146a* rs2910164 under the allelic in all and Asian populations and *AGO1* rs59561 in Asians under the recessive model. The former associations (SNPs rs2910164) were upgraded from weak to moderate (FPRP < 0.05); a publication bias may explain how the former associations were graded “ABC” overall based on Venice Criteria; these associations were thus rated as having moderate associations with LC risk because of a FPRP value <0.05. The latter variant (SNP rs59561) was not upgraded or downgraded based on FPRP value (0.05 < FPRP < 0.2); the criteria with amount of evidence may explain how this association was graded “BAA” overall based on Venice Criteria. However, this variant was conducted exclusively in Asians. One reason that concentrated on single (Asian) population could be the small sample size of this meta‐analysis, which made subgroup analyses challenging. Therefore, further expanding sample size and assessment on other ethnicities into this variant are necessary.

Seven associations were rated as being weakly associated with LC risk. Among these seven associations, *miRNA‐146a* rs2910164 under the recessive model and *miRNA‐499* rs3746444 under the dominant model were considered as being significant association with LC risk in all populations, while other five associations were significantly associated with LC risk in Asians, including *miRNA‐499* rs3746444 under the recessive model, *GEMIN4* rs7813 under the allelic model, *miRNA‐27a* rs895819 under the recessive model and *GEMIN4* rs910924 under the allelic and the dominant models. Of these variants, *GEMIN4* rs7813 increasing LC risk by 1.258‐fold in the meta‐analysis was well established, with an overall schema of ACA, indicating a high degree of heterogeneity that may explain how this variant was rated as weak based on Venice Criteria; no statistical data could be extracted for ethnicity subgroups, which could explain the heterogeneity in the data. We recommend subdividing populations by ethnicity to identify potential differences in the association between this variant and LC risk. Our study presented that *miRNA‐27a* rs895819 had no association with LC risk in all populations. Interestingly, we found that SNP rs895819 was significantly associated with LC risk in Asians (with a total sample of 4493), rather than in Caucasians (with a total sample of 4493). While sample size may be one factor affecting different associations between ethnicities, other factors such as methodology, LC subtypes, and environmental factors may also as account for variation in the data. Moreover, two variants, *miRNA‐146a* rs2910164 in all populations under the recessive model and *miRNA‐27a* rs895819 in Asians under the recessive model, were rated as weak, with an overall schema of ABC and BBC, respectively; the publication bias was main factor triggering the weak evidence for this variant, further investigations on these studies were recommended. Additionally, *GEMIN4* rs910924 under the allelic and the dominant models in Asians was rated as weakly associated with LC risk (with a total sample of 1051), after being downgraded from moderate to weak based on FPRP value (>0.2). Previous studies showed that setting different prior probabilities may make their results more noteworthy. Results therefore would be more convincing if different prior probabilities were assigned based on FPRP assay. Moreover, it may be necessary to further expand the sample size on current study and on other ethnicities into these variants, and then investigate these associations in greater depth.

Additionally, the associations stratified by different ethnicity or genetic models were usually inconsistent. For ethnicity, all studies were performed on a single ethnic group (Asian) except *miRNA‐146a* rs2910164 and *miRNA‐499* rs3746444 with 101 cases and 129 controls, respectively; the small sample size for non‐Asian race may make subgroup analyses challenging. For genetic models, three models were used for more comprehensive evaluation, while the existence of different genetic background such as age and gender about patients, subtypes of LC and environmental factors such as cigarette smoking were not be taken into consideration may present as sources of variation in the result. Further study on these factors is recommended.

Our study presented that five variants had no association with LC risk under all three genetic models, including *miRNA‐4293* rs12220909, *miRNA‐149* rs2292832, *miRNA‐608* rs4919510 and *miRNA‐26‐1* rs7372209 and *miRNA‐196a2* rs11614913; the former four SNPs were observed in a sample of approximately 4000 cases, at approximately 95% power to detect an OR of 1.15 in an allelic model for a variant with MAF of 20%. In addition, the last variant was observed in a sample of approximately 10 000 cases, at approximately 98% power to detect an OR of 1.15 in an allelic model for a variant with MAF of 20%. Therefore, these five variants may be not associated with LC risk. It is probable that further investigations evaluating these five variants will not yield fruitful results with LC risk if the sample size less than current study.

Some limitations should be considered to this study: (a) although available studies were searched widely, some publications may have been missed; (b) the excess of significant findings was not further evaluate due to insufficient data; (c) subgroup analyses were exclusively performed by ethnicity (only for Asians and Caucasians) and genetic models (only for allelic, dominant and recessive models), which may make the credibility of some results challenging, especially for weak evidence with small sample size; future evaluation with much larger sample size and other ethnicities may be necessary to confirm or refute these connections; (d) we only assessed the susceptibility/incidences of associations between SNPs in miRNA or biosynthesis genes and LC risk; the involvement of genetic polymorphisms as they contribute to cancer progression, metastasis, and drug resistance in LC was not evaluated due to insufficient data. Despite these limitations, we believe that this paper, which provides a comprehensive summary and evaluation of existing literature on the role of SNPs in miRNA or biosynthesis genes to LC, will be of value in informing future genetic studies.

In our study, Venice Criteria and FPRP test were introduced to evaluate the cumulative evidence of significant associations to increase the persuasion and accuracy of the final results. Two variants with four associations were rated as demonstrating strong evidence with risk of LC, three associations were moderate, and seven associations were weak. The results of SNPs in miRNAs or biosynthesis genes with LC risk may help us to get more potential target population for primary prevention. In summary, our study summarizes current literature on the SNPs in miRNA or biosynthesis genes architecture of LC susceptibility, and provides useful information for designing future studies aiming to evaluate SNPs in miRNA or biosynthesis genes factors for LC risk.

## CONFLICT OF INTEREST

The authors have no conflict of interest.

## Data Availability

Data derived from public domain resources. The data that support the findings of this study are available in PubMed, Medline, and Web of Science attached with reference number in manuscript. These data were derived from the following resources available in the public domain: PubMed, Medline, and Web of Science
