# Supplementary material for: Epidemiological evidence for associations between variants in microRNA or biosynthesis genes and lung cancer risk
Source: Cancer Med. 2020 Jan 7;9(5):1937–50. doi: 10.1002/cam4.2645 (PMC7050065; doi:10.1002/cam4.2645)
Supplement: Supplementary file 2 [file CAM4-9-1937-s002.docx]

**Supporting information to Figures**

**Figure S2.** presented the associations between *miR-196a2* rs11614913 and lung cancer risk under the different models, with forest plot, funnel plot, sensitive analysis.

**Supplementary Figure S2.1.** presented forest plot of association between miR-196a2 rs11614913 and lung cancer risk in all population under the Allelic model.

**Supplementary Figure S2.2.** presented forest plot of association between miR-196a2 rs11614913 and lung cancer risk under the Allelic model, stratified by ethnicity.

**Supplementary Figure S2.3.** presented funnel plot of association between miR-196a2 rs11614913 and lung cancer risk in all population under the Allelic model.

**Supplementary Figure S2.4.** presented sensitive analysis for association between miR-196a2 rs11614913 and lung cancer risk in all population under the Allelic model.

**Supplementary Figure S2.5.** presented forest plot of association between miR-196a2 rs11614913 and lung cancer risk in all population under the Dominant model.

**Supplementary Figure S2.6.** presented forest plot of association between miR-196a2 rs11614913 and lung cancer risk under the Dominant model, stratified by ethnicity.

**Supplementary Figure S2.7.** presented funnel plot of association between miR-196a2 rs11614913 and lung cancer risk in all population under the Dominant model.

**Supplementary Figure S2.8.** presented sensitive analysis for association between miR-196a2 rs11614913 and lung cancer risk in all population under the Dominant model.

**Supplementary Figure S2.9.** presented forest plot of association between miR-196a2 rs11614913 and lung cancer risk in all population under the Recessive model.

**Supplementary Figure S2.10.** presented forest plot of association between miR-196a2 rs11614913 and lung cancer risk under the Recessive model, stratified by ethnicity.

**Supplementary Figure S2.11.** presented funnel plot of association between miR-196a2 rs11614913 and lung cancer risk in all population under the Recessive model.

**Supplementary Figure S2.12.** presented sensitive analysis for association between miR-196a2 rs11614913 and lung cancer risk in all population under the Recessive model.
