# Supplementary material for: Epidemiological evidence for associations between variants in microRNA or biosynthesis genes and lung cancer risk
Source: Cancer Med. 2020 Jan 7;9(5):1937–50. doi: 10.1002/cam4.2645 (PMC7050065; doi:10.1002/cam4.2645)
Supplement: Supplementary file 3 [file CAM4-9-1937-s003.docx]

**Supporting information to Figures**

**Figure S3** presented the associations between *miR-4293* rs12220909 and lung cancer risk under the different models, with forest plot, funnel plot, sensitive analysis.

**Supplementary Figure S3.1.** presented forest plot of association between miR-4293 rs12220909 and lung cancer risk in Asian population under the Allelic model.

**Supplementary Figure S3.2.** presented funnel plot of association between miR-4293 rs12220909 and lung cancer risk in Asian population under the Allelic model.

**Supplementary Figure S3.3.** presented sensitive analysis for association between miR-4293 rs12220909 and lung cancer risk in Asian population under the Allelic model.

**Supplementary Figure S3.4.** presented forest plot of association between miR-4293 rs12220909 and lung cancer risk in Asian population under the Dominant model.

**Supplementary Figure S3.5.** presented funnel plot of association between miR-4293 rs12220909 and lung cancer risk in Asian population under the Dominant model.

**Supplementary Figure S3.6.** presented sensitive analysis for association between miR-4293 rs12220909 and lung cancer risk in Asian population under the Dominant model.

**Supplementary Figure S3.7.** presented forest plot of association between miR-4293 rs12220909 and lung cancer risk in Asian population under the Recessive model.

**Supplementary Figure S3.8.** presented funnel plot of association between miR-4293 rs12220909 and lung cancer risk in Asian population under the Recessive model.

**Supplementary Figure S3.9.** presented sensitive analysis for association between miR-4293 rs12220909 and lung cancer risk in Asian population under the Recessive model.
