# Supplementary material for: Epidemiological evidence for associations between variants in microRNA or biosynthesis genes and lung cancer risk
Source: Cancer Med. 2020 Jan 7;9(5):1937–50. doi: 10.1002/cam4.2645 (PMC7050065; doi:10.1002/cam4.2645)
Supplement: Supplementary file 4 [file CAM4-9-1937-s004.docx]

**Supporting information to Figures**

**Figure S4** presented the associations between *miR-149* rs2292832 and lung cancer risk under the different models, with forest plot, funnel plot, sensitive analysis.

**Supplementary Figure S4.1.** presented forest plot of association between miR-149 rs2292832 and lung cancer risk in all population under the Allelic model.

**Supplementary Figure S4.2.** presented forest plot of association between miR-149 rs2292832 and lung cancer risk under the Allelic model, stratified by ethnicity.

**Supplementary Figure S4.3.** presented funnel plot of association between miR-149 rs2292832 and lung cancer risk in all population under the Allelic model.

**Supplementary Figure S4.4.** presented sensitive analysis for association between miR-149 rs2292832 and lung cancer risk in all population under the Allelic model.

**Supplementary Figure S4.5.** presented forest plot of association between miR-149 rs2292832 and lung cancer risk in all population under the Dominant model.

**Supplementary Figure S4.6.** presented forest plot of association between miR-149 rs2292832 and lung cancer risk under the Dominant model, stratified by ethnicity.

**Supplementary Figure S4.7.** presented funnel plot of association between miR-149 rs2292832 and lung cancer risk in all population under the Dominant model.

**Supplementary Figure S4.8.** presented sensitive analysis for association between miR-149 rs2292832 and lung cancer risk in all population under the Dominant model.

**Supplementary Figure S4.9.** presented forest plot of association between miR-149 rs2292832 and lung cancer risk in all population under the Recessive model.

**Supplementary Figure S4.10** presented forest plot of association between miR-149 rs2292832 and lung cancer risk under the Recessive model, stratified by ethnicity.

**Supplementary Figure S4.11.** presented funnel plot of association between miR-149 rs2292832 and lung cancer risk in all population under the Recessive model.

**Supplementary Figure S4.12.** presented sensitive analysis for association between miR-149 rs2292832 and lung cancer risk in all population under the Recessive model.
