# Supplementary material for: Epidemiological evidence for associations between variants in microRNA or biosynthesis genes and lung cancer risk
Source: Cancer Med. 2020 Jan 7;9(5):1937–50. doi: 10.1002/cam4.2645 (PMC7050065; doi:10.1002/cam4.2645)
Supplement: Supplementary file 5 [file CAM4-9-1937-s005.docx]

**Supporting information to Figures**

**Figure S5** presented the associations between miR-499 rs3746444 and lung cancer risk under the different models, with forest plot, funnel plot, sensitive analysis

.

**Supplementary Figure S5.1.** presented forest plot of association between miR-499 rs3746444 and lung cancer risk in all population under the Allelic model

**Supplementary Figure S5.2.** presented forest plot of association between miR-499 rs3746444 and lung cancer risk under the Allelic model, stratified by ethnicity.

**Supplementary Figure S5.3.** presented funnel plot of association between miR-499 rs3746444 and lung cancer risk in all population under the Allelic model.

**Supplementary Figure S5.4.** presented sensitive analysis for association between miR-499 rs3746444 and lung cancer risk in all population under the Allelic model.

**Supplementary Figure S5.5.** presented forest plot of association between miR-499 rs3746444 and lung cancer risk in all population under the Dominant model.

**Supplementary Figure S5.6.** presented forest plot of association between miR-499 rs3746444 and lung cancer risk under the Dominant model, stratified by ethnicity.

**Supplementary Figure S5.7.** presented funnel plot of association between miR-499 rs3746444 and lung cancer risk in all population under the Dominant model.

**Supplementary Figure S5.8.** presented sensitive analysis for association between miR-499 rs3746444 and lung cancer risk in all population under the Dominant model.

**Supplementary Figure S5.9.** presented forest plot of association between miR-499 rs3746444 and lung cancer risk in all population under the Recessive model.

**Supplementary Figure S5.10.** presented forest plot of association between miR-499 rs3746444 and lung cancer risk under the Recessive model, stratified by ethnicity.

**Supplementary Figure S5.11.** Funnel plot of association between miR-499 rs3746444 and lung cancer risk in all population under the Recessive model.

**Supplementary Figure S5.12.** presented sensitive analysis for association between miR-499 rs3746444 and lung cancer risk in all population under the Recessive model.
