# Supplementary material for: Epidemiological evidence for associations between variants in microRNA or biosynthesis genes and lung cancer risk
Source: Cancer Med. 2020 Jan 7;9(5):1937–50. doi: 10.1002/cam4.2645 (PMC7050065; doi:10.1002/cam4.2645)
Supplement: Supplementary file 11 [file CAM4-9-1937-s011.docx]

**Supporting information to Figures**

**Figure S11** presented the associations between *GEMIN4* rs910924 and lung cancer risk under the different models, with forest plot, funnel plot, sensitive analysis.

**Supplementary Figure S11.1.** presented forest plot of association between GEMIN4 rs910924 and lung cancer risk in Asian population under the Allelic model.

**Supplementary Figure S11.2.** presented funnel plot of association between GEMIN4 rs910924 and lung cancer risk in Asian population under the Allelic model.

**Supplementary Figure S11.3.** presented sensitive analysis for association between GEMIN4 rs910924 and lung cancer risk in Asian population under the Allelic model.

**Supplementary Figure S11.4.** presented forest plot of association between GEMIN4 rs910924 and lung cancer risk in Asian population under the Dominant model.

**Supplementary Figure S11.5.** presented funnel plot of association between GEMIN4 rs910924 and lung cancer risk in Asian population under the Dominant model.

**Supplementary Figure S11.6.** presented sensitive analysis for association between GEMIN4 rs910924 and lung cancer risk in Asian population under the Dominant model.

**Supplementary Figure S11.7.** presented forest plot of association between GEMIN4 rs910924 and lung cancer risk in Asian population under the Recessive model.

**Supplementary Figure S11.8.** presented funnel plot of association between GEMIN4 rs910924 and lung cancer risk in Asian population under the Recessive model.

**Supplementary Figure S11.9.** presented sensitive analysis for association between GEMIN4 rs910924 and lung cancer risk in Asian population under the Recessive model.
