# Supplementary material for: Epidemiological evidence for associations between variants in microRNA or biosynthesis genes and lung cancer risk
Source: Cancer Med. 2020 Jan 7;9(5):1937–50. doi: 10.1002/cam4.2645 (PMC7050065; doi:10.1002/cam4.2645)
Supplement: Supplementary file 13 [file CAM4-9-1937-s013.docx]

**Supplementary Table S2: Newcastle Ottawa Scale (NOS) of case-control studies was used to evaluate the quality for each eligible study.**

| **Author, year^(Ref)^** | **Selection** | | | | **Comparability** | **EXPOSURE** | | | **Overall quality** |
| --- | --- | --- | --- | --- | --- | --- | --- | --- | --- |
|  | **Is the Case Definition Adequate?** | **Representativeness of**  **the Cases** | **Selection of**  **Controls** | **Definition**  **of**  **Controls** | **Comparability of Cases and Controls on the Basis of the Design or Analysis** | **Ascertainment of**  **Exposure** | **Same method of ascertainment for cases and controls** | **Non-Response Rate** |  |
| Liu Z, 2018^(1)^ | ★ | ★ | ★ | ★ | ★☆ | ★ | ★ | ☆ | **7** |
| Yin Z, 2017^(2)^ | ★ | ★ | ★ | ☆ | ★☆ | ★ | ★ | ☆ | **6** |
| Fan L, 2017^(3)^ | ★ | ★ | ★ | ★ | ★☆ | ★ | ★ | ☆ | **7** |
| Yin Z, 2017^(4)^ | ★ | ★ | ★ | ★ | ★☆ | ★ | ★ | ☆ | **7** |
| Li H, 2016^(5)^ | ★ | ★ | ★ | ★ | ☆☆ | ★ | ★ | ☆ | **6** |
| Fang X, 2016^(6)^ | ★ | ★ | ★ | ★ | ★☆ | ★ | ★ | ☆ | **7** |
| Li D, 2016^(7)^ | ★ | ★ | ★ | ★ | ★☆ | ★ | ★ | ☆ | **7** |
| Yin Z, 2016^(8)^ | ★ | ★ | ★ | ★ | ☆☆ | ★ | ★ | ☆ | **6** |
| Yin Z, 2016^(9)^ | ★ | ★ | ★ | ★ | ★☆ | ★ | ★ | ☆ | **7** |
| Sodhi,K, 2015^(10)^ | ★ | ★ | ★ | ★ | ★☆ | ★ | ★ | ☆ | **7** |
| Ma,J.Y, 2015^(11)^ | ★ | ★ | ★ | ★ | ★☆ | ★ | ★ | ☆ | **7** |
| Jia Y, 2014^(12)^ | ★ | ★ | ★ | ★ | ★☆ | ★ | ★ | ☆ | **7** |
| Jeon HS, 2014^(13)^ | ★ | ★ | ★ | ★ | ★☆ | ★ | ★ | ☆ | **7** |
| Vinci S, 2012 ^(14)^ | ★ | ★ | ★ | ★ | ★☆ | ★ | ★ | ☆ | **7** |
| Hong YS, 2011^(15)^ | ★ | ★ | ★ | ★ | ★☆ | ★ | ★ | ☆ | **7** |
| Kim JS, 2010^(16)^ | ★ | ★ | ★ | ★ | ★☆ | ★ | ★ | ☆ | **7** |
| Tian T, 2009^(17)^ | ★ | ★ | ★ | ★ | ★☆ | ★ | ★ | ☆ | **7** |
| Yin Z, 2015^(18)^ | ★ | ★ | ★ | ★ | ★☆ | ★ | ★ | ☆ | **7** |
| Kim MJ, 2010^(19)^ | ★ | ★ | ★ | ★ | ★☆ | ★ | ★ | ☆ | **7** |
| Xie K, 2017^(20)^ | ★ | ★ | ★ | ★ | ★☆ | ★ | ★ | ☆ | **7** |

No.of studies: **20**

Overall quality of all study: 137

**Average: 6.85**

References:

1. [Liu Z](https://www.ncbi.nlm.nih.gov/pubmed/?term=Liu Z[Author]&cauthor=true&cauthor_uid=30271206), [Lin Y](https://www.ncbi.nlm.nih.gov/pubmed/?term=Lin Y[Author]&cauthor=true&cauthor_uid=30271206), [Kang S](https://www.ncbi.nlm.nih.gov/pubmed/?term=Kang S[Author]&cauthor=true&cauthor_uid=30271206), [Xu Q](https://www.ncbi.nlm.nih.gov/pubmed/?term=Xu Q[Author]&cauthor=true&cauthor_uid=30271206), [Xiong W](https://www.ncbi.nlm.nih.gov/pubmed/?term=Xiong W[Author]&cauthor=true&cauthor_uid=30271206), [Cai L](https://www.ncbi.nlm.nih.gov/pubmed/?term=Cai L[Author]&cauthor=true&cauthor_uid=30271206), et al. miR-300 rs12894467 polymorphism may be associated with susceptibility to primary lung cancer in the Chinese Han population. [Cancer Manag Res.](https://www.ncbi.nlm.nih.gov/pubmed/?term=30271206) 2018;10:3579-3588.
2. [Yin Z](https://www.ncbi.nlm.nih.gov/pubmed/?term=Yin Z[Author]&cauthor=true&cauthor_uid=29050330), [Cui Z](https://www.ncbi.nlm.nih.gov/pubmed/?term=Cui Z[Author]&cauthor=true&cauthor_uid=29050330), [Ren Y](https://www.ncbi.nlm.nih.gov/pubmed/?term=Ren Y[Author]&cauthor=true&cauthor_uid=29050330), [Xia L](https://www.ncbi.nlm.nih.gov/pubmed/?term=Xia L[Author]&cauthor=true&cauthor_uid=29050330), [Li H](https://www.ncbi.nlm.nih.gov/pubmed/?term=Li H[Author]&cauthor=true&cauthor_uid=29050330), [Zhou B](https://www.ncbi.nlm.nih.gov/pubmed/?term=Zhou B[Author]&cauthor=true&cauthor_uid=29050330), et al. MiR-196a2 and lung cancer in Chinese non-smoking females: a genetic association study and expression analysis. [Oncotarget.](https://www.ncbi.nlm.nih.gov/pubmed/?term=29050330) 2017;8(41):70890-70898.
3. [Fan L](https://www.ncbi.nlm.nih.gov/pubmed/?term=Fan L[Author]&cauthor=true&cauthor_uid=28410417), [Chen L](https://www.ncbi.nlm.nih.gov/pubmed/?term=Chen L[Author]&cauthor=true&cauthor_uid=28410417), [Ni X](https://www.ncbi.nlm.nih.gov/pubmed/?term=Ni X[Author]&cauthor=true&cauthor_uid=28410417), [Guo S](https://www.ncbi.nlm.nih.gov/pubmed/?term=Guo S[Author]&cauthor=true&cauthor_uid=28410417), [Zhou Y](https://www.ncbi.nlm.nih.gov/pubmed/?term=Zhou Y[Author]&cauthor=true&cauthor_uid=28410417), [Wang C](https://www.ncbi.nlm.nih.gov/pubmed/?term=Wang C[Author]&cauthor=true&cauthor_uid=28410417), et al. Genetic variant of miR-4293 rs12220909 is associated with susceptibility to non-small cell lung cancer in a Chinese Han population. [PLoS One.](https://www.ncbi.nlm.nih.gov/pubmed/?term=28410417) 2017;12(4):e0175666.
4. [Yin Z](https://www.ncbi.nlm.nih.gov/pubmed/?term=Yin Z[Author]&cauthor=true&cauthor_uid=27911870), [Cui Z](https://www.ncbi.nlm.nih.gov/pubmed/?term=Cui Z[Author]&cauthor=true&cauthor_uid=27911870), [Ren Y](https://www.ncbi.nlm.nih.gov/pubmed/?term=Ren Y[Author]&cauthor=true&cauthor_uid=27911870), [Xia L](https://www.ncbi.nlm.nih.gov/pubmed/?term=Xia L[Author]&cauthor=true&cauthor_uid=27911870), [Li H](https://www.ncbi.nlm.nih.gov/pubmed/?term=Li H[Author]&cauthor=true&cauthor_uid=27911870), [Zhou B](https://www.ncbi.nlm.nih.gov/pubmed/?term=Zhou B[Author]&cauthor=true&cauthor_uid=27911870). MiR-146a polymorphism correlates with lung cancer risk in Chinese nonsmoking females. [Oncotarget.](https://www.ncbi.nlm.nih.gov/pubmed/?term=27911870) 2017;8(2):2275-2283.
5. [Li H](https://www.ncbi.nlm.nih.gov/pubmed/?term=Li H[Author]&cauthor=true&cauthor_uid=27685326), [Ren Y](https://www.ncbi.nlm.nih.gov/pubmed/?term=Ren Y[Author]&cauthor=true&cauthor_uid=27685326), [Xia L](https://www.ncbi.nlm.nih.gov/pubmed/?term=Xia L[Author]&cauthor=true&cauthor_uid=27685326), [Qu R](https://www.ncbi.nlm.nih.gov/pubmed/?term=Qu R[Author]&cauthor=true&cauthor_uid=27685326), [Kong L](https://www.ncbi.nlm.nih.gov/pubmed/?term=Kong L[Author]&cauthor=true&cauthor_uid=27685326), [Yin Z](https://www.ncbi.nlm.nih.gov/pubmed/?term=Yin Z[Author]&cauthor=true&cauthor_uid=27685326), et al. Association of MicroRNA-149 Polymorphism with Lung Cancer Risk in Chinese Non-Smoking Female: A Case-Control Study. [PLoS One.](https://www.ncbi.nlm.nih.gov/pubmed/?term=27685326) 2016;11(9):e0163626.
6. [Fang X](https://www.ncbi.nlm.nih.gov/pubmed/?term=Fang X[Author]&cauthor=true&cauthor_uid=27669275), [Yin Z](https://www.ncbi.nlm.nih.gov/pubmed/?term=Yin Z[Author]&cauthor=true&cauthor_uid=27669275), [Li X](https://www.ncbi.nlm.nih.gov/pubmed/?term=Li X[Author]&cauthor=true&cauthor_uid=27669275), [Xia L](https://www.ncbi.nlm.nih.gov/pubmed/?term=Xia L[Author]&cauthor=true&cauthor_uid=27669275), [Zhou B](https://www.ncbi.nlm.nih.gov/pubmed/?term=Zhou B[Author]&cauthor=true&cauthor_uid=27669275). Polymorphisms in GEMIN4 and AGO1 Genes Are Associated with the Risk of Lung Cancer: A Case-Control Study in Chinese Female Non-Smokers. [Int J Environ Res Public Health.](https://www.ncbi.nlm.nih.gov/pubmed/?term=27669275) 2016;13(10). pii: E939.
7. [Li D](https://www.ncbi.nlm.nih.gov/pubmed/?term=Li D[Author]&cauthor=true&cauthor_uid=27232940), [Zhu G](https://www.ncbi.nlm.nih.gov/pubmed/?term=Zhu G[Author]&cauthor=true&cauthor_uid=27232940), [Di H](https://www.ncbi.nlm.nih.gov/pubmed/?term=Di H[Author]&cauthor=true&cauthor_uid=27232940), [Li H](https://www.ncbi.nlm.nih.gov/pubmed/?term=Li H[Author]&cauthor=true&cauthor_uid=27232940), [Liu X](https://www.ncbi.nlm.nih.gov/pubmed/?term=Liu X[Author]&cauthor=true&cauthor_uid=27232940), [Zhao M](https://www.ncbi.nlm.nih.gov/pubmed/?term=Zhao M[Author]&cauthor=true&cauthor_uid=27232940), et al. Associations between genetic variants located in mature microRNAs and risk of lung cancer. [Oncotarget.](https://www.ncbi.nlm.nih.gov/pubmed/?term=27232940) 2016; 7(27): 41715-41724.
8. [Yin Z](https://www.ncbi.nlm.nih.gov/pubmed/?term=Yin Z[Author]&cauthor=true&cauthor_uid=26973201), [Cui Z](https://www.ncbi.nlm.nih.gov/pubmed/?term=Cui Z[Author]&cauthor=true&cauthor_uid=26973201), [Ren Y](https://www.ncbi.nlm.nih.gov/pubmed/?term=Ren Y[Author]&cauthor=true&cauthor_uid=26973201), [Xia L](https://www.ncbi.nlm.nih.gov/pubmed/?term=Xia L[Author]&cauthor=true&cauthor_uid=26973201), [Wang Q](https://www.ncbi.nlm.nih.gov/pubmed/?term=Wang Q[Author]&cauthor=true&cauthor_uid=26973201), [Zhang Y](https://www.ncbi.nlm.nih.gov/pubmed/?term=Zhang Y[Author]&cauthor=true&cauthor_uid=26973201), et al. Association between polymorphisms in pre-miRNA genes and risk of lung cancer in a Chinese non-smoking female population. [Lung Cancer.](https://www.ncbi.nlm.nih.gov/pubmed/?term=26973201" \o "Lung cancer (Amsterdam, Netherlands).) 2016;94:15-21.
9. [Yin Z](https://www.ncbi.nlm.nih.gov/pubmed/?term=Yin Z[Author]&cauthor=true&cauthor_uid=26855588), [Li H](https://www.ncbi.nlm.nih.gov/pubmed/?term=Li H[Author]&cauthor=true&cauthor_uid=26855588), [Cui Z](https://www.ncbi.nlm.nih.gov/pubmed/?term=Cui Z[Author]&cauthor=true&cauthor_uid=26855588), [Ren Y](https://www.ncbi.nlm.nih.gov/pubmed/?term=Ren Y[Author]&cauthor=true&cauthor_uid=26855588), [Li X](https://www.ncbi.nlm.nih.gov/pubmed/?term=Li X[Author]&cauthor=true&cauthor_uid=26855588), [Wu W](https://www.ncbi.nlm.nih.gov/pubmed/?term=Wu W[Author]&cauthor=true&cauthor_uid=26855588), et al. Polymorphisms in pre-miRNA genes and cooking oil fume exposure as well as their interaction on the risk of lung cancer in a Chinese nonsmoking female population. [Onco Targets Ther.](https://www.ncbi.nlm.nih.gov/pubmed/?term=26855588) 2016;9:395-401.
10. [Sodhi KK](https://www.ncbi.nlm.nih.gov/pubmed/?term=Sodhi KK[Author]&cauthor=true&cauthor_uid=26235181), [Bahl C](https://www.ncbi.nlm.nih.gov/pubmed/?term=Bahl C[Author]&cauthor=true&cauthor_uid=26235181), [Singh N](https://www.ncbi.nlm.nih.gov/pubmed/?term=Singh N[Author]&cauthor=true&cauthor_uid=26235181), [Behera D](https://www.ncbi.nlm.nih.gov/pubmed/?term=Behera D[Author]&cauthor=true&cauthor_uid=26235181), [Sharma S](https://www.ncbi.nlm.nih.gov/pubmed/?term=Sharma S[Author]&cauthor=true&cauthor_uid=26235181). Functional genetic variants in pre-miR-146a and 196a2 genes are associated with risk of lung cancer in North Indians. [Future Oncol.](https://www.ncbi.nlm.nih.gov/pubmed/?term=26235181" \o "Future oncology (London, England).) 2015;11(15):2159-2173.
11. [Ma JY](https://www.ncbi.nlm.nih.gov/pubmed/?term=Ma JY[Author]&cauthor=true&cauthor_uid=25773791), [Yan HJ](https://www.ncbi.nlm.nih.gov/pubmed/?term=Yan HJ[Author]&cauthor=true&cauthor_uid=25773791), [Yang ZH](https://www.ncbi.nlm.nih.gov/pubmed/?term=Yang ZH[Author]&cauthor=true&cauthor_uid=25773791), [Gu W](https://www.ncbi.nlm.nih.gov/pubmed/?term=Gu W[Author]&cauthor=true&cauthor_uid=25773791). Rs895819 within miR-27a might be involved in development of non small cell lung cancer in the Chinese Han population. [Asian Pac J Cancer Prev.](https://www.ncbi.nlm.nih.gov/pubmed/?term=25773791) 2015;16(5):1939-1944.
12. [Jia Y](https://www.ncbi.nlm.nih.gov/pubmed/?term=Jia Y[Author]&cauthor=true&cauthor_uid=25154761), [Zang A](https://www.ncbi.nlm.nih.gov/pubmed/?term=Zang A[Author]&cauthor=true&cauthor_uid=25154761), [Shang Y](https://www.ncbi.nlm.nih.gov/pubmed/?term=Shang Y[Author]&cauthor=true&cauthor_uid=25154761), [Yang H](https://www.ncbi.nlm.nih.gov/pubmed/?term=Yang H[Author]&cauthor=true&cauthor_uid=25154761), [Song Z](https://www.ncbi.nlm.nih.gov/pubmed/?term=Song Z[Author]&cauthor=true&cauthor_uid=25154761), [Wang Z](https://www.ncbi.nlm.nih.gov/pubmed/?term=Wang Z[Author]&cauthor=true&cauthor_uid=25154761), et al. MicroRNA-146a rs2910164 polymorphism is associated with susceptibility to non-small cell lung cancer in the Chinese population. [Med Oncol.](https://www.ncbi.nlm.nih.gov/pubmed/?term=25154761" \o "Medical oncology (Northwood, London, England).) 2014 ;31(10):194.
13. [Jeon HS](https://www.ncbi.nlm.nih.gov/pubmed/?term=Jeon HS[Author]&cauthor=true&cauthor_uid=24144839), [Lee YH](https://www.ncbi.nlm.nih.gov/pubmed/?term=Lee YH[Author]&cauthor=true&cauthor_uid=24144839), [Lee SY](https://www.ncbi.nlm.nih.gov/pubmed/?term=Lee SY[Author]&cauthor=true&cauthor_uid=24144839), [Jang JA](https://www.ncbi.nlm.nih.gov/pubmed/?term=Jang JA[Author]&cauthor=true&cauthor_uid=24144839), [Choi YY](https://www.ncbi.nlm.nih.gov/pubmed/?term=Choi YY[Author]&cauthor=true&cauthor_uid=24144839), [Yoo SS](https://www.ncbi.nlm.nih.gov/pubmed/?term=Yoo SS[Author]&cauthor=true&cauthor_uid=24144839), et al. A common polymorphism in pre-microRNA-146a is associated with lung cancer risk in a Korean population. [Gene.](https://www.ncbi.nlm.nih.gov/pubmed/?term=24144839) 2014;534(1):66-71.
14. [Vinci S](https://www.ncbi.nlm.nih.gov/pubmed/?term=Vinci S[Author]&cauthor=true&cauthor_uid=21902575), [Gelmini S](https://www.ncbi.nlm.nih.gov/pubmed/?term=Gelmini S[Author]&cauthor=true&cauthor_uid=21902575), [Pratesi N](https://www.ncbi.nlm.nih.gov/pubmed/?term=Pratesi N[Author]&cauthor=true&cauthor_uid=21902575), [Conti S](https://www.ncbi.nlm.nih.gov/pubmed/?term=Conti S[Author]&cauthor=true&cauthor_uid=21902575), [Malentacchi F](https://www.ncbi.nlm.nih.gov/pubmed/?term=Malentacchi F[Author]&cauthor=true&cauthor_uid=21902575), [Simi L](https://www.ncbi.nlm.nih.gov/pubmed/?term=Simi L[Author]&cauthor=true&cauthor_uid=21902575), et al. Genetic variants in miR-146a, miR-149, miR-196a2, miR-499 and their influence on relative expression in lung cancers. [Clin Chem Lab Med.](https://www.ncbi.nlm.nih.gov/pubmed/?term=21902575) 2011;49(12):2073-2080.
15. [Hong YS](https://www.ncbi.nlm.nih.gov/pubmed/?term=Hong YS[Author]&cauthor=true&cauthor_uid=21617338), [Kang HJ](https://www.ncbi.nlm.nih.gov/pubmed/?term=Kang HJ[Author]&cauthor=true&cauthor_uid=21617338), [Kwak JY](https://www.ncbi.nlm.nih.gov/pubmed/?term=Kwak JY[Author]&cauthor=true&cauthor_uid=21617338), [Park BL](https://www.ncbi.nlm.nih.gov/pubmed/?term=Park BL[Author]&cauthor=true&cauthor_uid=21617338), [You CH](https://www.ncbi.nlm.nih.gov/pubmed/?term=You CH[Author]&cauthor=true&cauthor_uid=21617338), [Kim YM](https://www.ncbi.nlm.nih.gov/pubmed/?term=Kim YM[Author]&cauthor=true&cauthor_uid=21617338), et al. Association between microRNA196a2 rs11614913 genotypes and the risk of non-small cell lung cancer in Korean population. [J Prev Med Public Health.](https://www.ncbi.nlm.nih.gov/pubmed/?term=21617338) 2011;44(3):125-130.
16. [Kim JS](https://www.ncbi.nlm.nih.gov/pubmed/?term=Kim JS[Author]&cauthor=true&cauthor_uid=20721975), [Choi YY](https://www.ncbi.nlm.nih.gov/pubmed/?term=Choi YY[Author]&cauthor=true&cauthor_uid=20721975), [Jin G](https://www.ncbi.nlm.nih.gov/pubmed/?term=Jin G[Author]&cauthor=true&cauthor_uid=20721975), [Kang HG](https://www.ncbi.nlm.nih.gov/pubmed/?term=Kang HG[Author]&cauthor=true&cauthor_uid=20721975), [Choi JE](https://www.ncbi.nlm.nih.gov/pubmed/?term=Choi JE[Author]&cauthor=true&cauthor_uid=20721975), [Jeon HS](https://www.ncbi.nlm.nih.gov/pubmed/?term=Jeon HS[Author]&cauthor=true&cauthor_uid=20721975), et al. Association of a common AGO1 variant with lung cancer risk: a two-stage case-control study. [Mol Carcinog.](https://www.ncbi.nlm.nih.gov/pubmed/?term=20721975) 2010;49(10):913-921.
17. [Tian T](https://www.ncbi.nlm.nih.gov/pubmed/?term=Tian T[Author]&cauthor=true&cauthor_uid=19293314), [Shu Y](https://www.ncbi.nlm.nih.gov/pubmed/?term=Shu Y[Author]&cauthor=true&cauthor_uid=19293314), [Chen J](https://www.ncbi.nlm.nih.gov/pubmed/?term=Chen J[Author]&cauthor=true&cauthor_uid=19293314), [Hu Z](https://www.ncbi.nlm.nih.gov/pubmed/?term=Hu Z[Author]&cauthor=true&cauthor_uid=19293314), [Xu L](https://www.ncbi.nlm.nih.gov/pubmed/?term=Xu L[Author]&cauthor=true&cauthor_uid=19293314), [Jin G](https://www.ncbi.nlm.nih.gov/pubmed/?term=Jin G[Author]&cauthor=true&cauthor_uid=19293314), et al. A functional genetic variant in microRNA-196a2 is associated with increased susceptibility of lung cancer in Chinese. [Cancer Epidemiol Biomarkers Prev.](https://www.ncbi.nlm.nih.gov/pubmed/?term=19293314) 2009;18(4):1183-1187.
18. [Yin Z](https://www.ncbi.nlm.nih.gov/pubmed/?term=Yin Z[Author]&cauthor=true&cauthor_uid=26083623), [Cui Z](https://www.ncbi.nlm.nih.gov/pubmed/?term=Cui Z[Author]&cauthor=true&cauthor_uid=26083623), [Guan P](https://www.ncbi.nlm.nih.gov/pubmed/?term=Guan P[Author]&cauthor=true&cauthor_uid=26083623), [Li X](https://www.ncbi.nlm.nih.gov/pubmed/?term=Li X[Author]&cauthor=true&cauthor_uid=26083623), [Wu W](https://www.ncbi.nlm.nih.gov/pubmed/?term=Wu W[Author]&cauthor=true&cauthor_uid=26083623), [Ren Y](https://www.ncbi.nlm.nih.gov/pubmed/?term=Ren Y[Author]&cauthor=true&cauthor_uid=26083623), et al. Interaction between Polymorphisms in Pre-MiRNA Genes and Cooking Oil Fume Exposure on the Risk of Lung Cancer in Chinese Non-Smoking Female Population. [PLoS One.](https://www.ncbi.nlm.nih.gov/pubmed/?term=26083623) 2015;10(6):e0128572.
19. [Kim MJ](https://www.ncbi.nlm.nih.gov/pubmed/?term=Kim MJ[Author]&cauthor=true&cauthor_uid=20466450), [Yoo SS](https://www.ncbi.nlm.nih.gov/pubmed/?term=Yoo SS[Author]&cauthor=true&cauthor_uid=20466450), [Choi YY](https://www.ncbi.nlm.nih.gov/pubmed/?term=Choi YY[Author]&cauthor=true&cauthor_uid=20466450), [Park JY](https://www.ncbi.nlm.nih.gov/pubmed/?term=Park JY[Author]&cauthor=true&cauthor_uid=20466450). A functional polymorphism in the pre-microRNA-196a2 and the risk of lung cancer in a Korean population. [Lung Cancer.](https://www.ncbi.nlm.nih.gov/pubmed/?term=20466450" \o "Lung cancer (Amsterdam, Netherlands).) 2010; 69(1): 127-129.
20. [Xie K](https://www.ncbi.nlm.nih.gov/pubmed/?term=Xie K[Author]&cauthor=true&cauthor_uid=28510306), [Chen M](https://www.ncbi.nlm.nih.gov/pubmed/?term=Chen M[Author]&cauthor=true&cauthor_uid=28510306), [Zhu M](https://www.ncbi.nlm.nih.gov/pubmed/?term=Zhu M[Author]&cauthor=true&cauthor_uid=28510306), [Wang C](https://www.ncbi.nlm.nih.gov/pubmed/?term=Wang C[Author]&cauthor=true&cauthor_uid=28510306), [Qin N](https://www.ncbi.nlm.nih.gov/pubmed/?term=Qin N[Author]&cauthor=true&cauthor_uid=28510306), [Liang C](https://www.ncbi.nlm.nih.gov/pubmed/?term=Liang C[Author]&cauthor=true&cauthor_uid=28510306), et al. A polymorphism in miR-1262 regulatory region confers the risk of lung cancer in Chinese population. [Int J Cancer.](https://www.ncbi.nlm.nih.gov/pubmed/?term=28510306) 2017;141(5):958-966.
