# Supplementary material for: Epidemiological evidence for associations between variants in microRNA or biosynthesis genes and lung cancer risk
Source: Cancer Med. 2020 Jan 7;9(5):1937–50. doi: 10.1002/cam4.2645 (PMC7050065; doi:10.1002/cam4.2645)
Supplement: Supplementary file 14 [file CAM4-9-1937-s014.docx]

**Supplementary Table S3: Supplementary notes for genetic model with the complete data structure of genetic polymorphism study**

| Genotype amount | | | |
| --- | --- | --- | --- |
| Genotype type | A A | A B | BB |
| Case group | a_n_ | b_n_ | c_n_ |
| Control group | d_n_ | e_n_ | f_n_ |

AA:Wild homozygous AB: Heterozygous mutant BB: Mutant homozygous

n: indicating the Nth study.

For a SNP, two alleles, A and B, could be presented. Specifically, A was considered as wild type, meanwhile, B was considered mutant type. Therefore, there may be three genotypes, AA, AB, BB, respectively, in population. Suppose there were there three genotypes of the subjects, we could assign a,b,c to AA, AB, BB in case group, and d,e,f to AA, AB, BB in control group, respectively. The table above could offer additional explanation.

In meta-analysis for SNPs, polygenic model was used to decrease probabilities of type I error. The following genetic models may be used in our study: 1) Allelic model (i.e. B vs A); 2) Dominant model (BB+BA vs AA); 3) Recessive model (BB vs BA+AA) . Specifically, the allelic model was used first, and the rest models were also used when allelic model was not usable.
