# Supplementary material for: Epidemiological evidence for associations between variants in microRNA or biosynthesis genes and lung cancer risk
Source: Cancer Med. 2020 Jan 7;9(5):1937–50. doi: 10.1002/cam4.2645 (PMC7050065; doi:10.1002/cam4.2645)
Supplement: Supplementary file 15 [file CAM4-9-1937-s015.docx]

**Supplementary Table 4: Genetic associations with lung cancer risk for all 11 variants analyzed in main meta-analyses under the different genetic models.**

| **Gene** | **Variant** | **allelics** | **Ethnicity** | **Number Evaluation** | |  |  |  | **Risk of Meta-Analysis** | | | | **Venice Criteria** | **Venice Criteria Grade** | **Amount of Evidence** | | **Replication** | **Protection from Bias** | | **First Study** | **Reason for bias Exemption** | **Deviation from HWE** | **Pegger** | **Pbegger** | **Low OR** | **FPRP** | **Credibility of Evidence** |
| --- | --- | --- | --- | --- | --- | --- | --- | --- | --- | --- | --- | --- | --- | --- | --- | --- | --- | --- | --- | --- | --- | --- | --- | --- | --- | --- | --- |
|  |  |  |  | **studies** | **Cases**  **/controls** | **Genetic**  **Models** | **MAF^1^** | **effect model** | **OR (95%CI)** | **I^2^** | **P_value_** | **P_Q_** |  |  | **Nminor** | **Grade** |  | **Grade** | **Reasons for Bias** |  |  |  |  |  |  |  |  |
| **miR-146a** | **rs2910164** | **C>G** | **All** | **9** | **5888/5894** | **Allelic** |  | **fixed** | **0.886 (0.841-0.934)** | **34.4** | **<0.001** | **0.143** | **ABC** | **Weak** | **11220** | **A** | **B** | **C** | **publication bias** | **No** | **No** | **No** | **0.057** | **0.348** | **No** | **<0.001** | **Moderate** |
|  |  |  |  |  |  | **Dominant** |  | **fixed** | **0.835 (0.769-0.906)** | **0** | **<0.001** | **0.512** | **AAA** | **Strong** | **8400** | **A** | **A** | **A** |  | **No** | **No** | **No** | **0.439** | **0.466** | **No** | **<0.001** | **Strong** |
|  |  |  |  |  |  | **Recessive** |  | **fixed** | **0.875 (0.801-0.956)** | **25.2** | **0.003** | **0.219** | **ABC** | **Weak** | **2820** | **A** | **B** | **C** | **publication bias** | **No** | **No** | **No** | **0.043** | **0.048** | **No** | **0.124** | **Weak** |
|  |  |  | **Asian** | **8** | **5787/5765** | **Allelic** |  | **fixed** | **0.889 (0.843-0.937)** | **37.6** | **<0.001** | **0.13** | **ABC** | **Weak** | **10873** | **A** | **B** | **C** | **publication bias** | **No** | **No** | **No** | **0.094** | **0.536** | **No** | **<0.001** | **Moderate** |
|  |  |  |  |  |  | **Dominant** |  | **fixed** | **0.834 (0.768-0.905)** | **2.1** | **<0.001** | **0.414** | **AAA** | **Strong** | **8190** | **A** | **A** | **A** |  | **No** | **No** | **No** | **0.272** | **0.386** | **No** | **<0.001** | **Strong** |
|  |  |  |  |  |  | **Recessive** |  | **fixed** | **0.885 (0.809-0.968)** | **17.8** | **0.008** | **0.29** | **AAA** | **Strong** | **2703** | **A** | **A** | **A** |  | **No** | **No** | **No** | **0.13** | **0.174** | **No** | **0.126** | **Strong** |
|  |  |  | **Caucasian** | **1** | **101/129** | **Allelic** |  | **fixed** | **0.723 (0.482-1.084)** | **Na** | **Na** | **Na** |  |  |  |  |  |  |  |  |  |  |  |  |  |  |  |
|  |  |  |  |  |  | **Dominant** |  | **fixed** | **0.953 (0.379-2.396)** | **Na** | **Na** | **Na** |  |  |  |  |  |  |  |  |  |  |  |  |  |  |  |
|  |  |  |  |  |  | **Recessive** |  | **fixed** | **0.592 (0.350-1.001)** | **Na** | **Na** | **Na** |  |  |  |  |  |  |  |  |  |  |  |  |  |  |  |
| **miR-196a2** | **rs11614913** | **C>T** | **All** | **8** | **4736/4851** | **Allelic** |  | **random** | **0.952 (0.848-1.070)** | **Na** | **Na** | **Na** |  |  |  |  |  |  |  |  |  |  |  |  |  |  |  |
|  |  |  |  |  |  | **Dominant** |  | **random** | **0.985 (0.835-1.162)** | **62.3** | **0.854** | **0.01** |  |  |  |  |  |  |  |  |  |  |  |  |  |  |  |
|  |  |  |  |  |  | **Recessive** |  | **random** | **0.878 (0.710-1.087)** | **76.3** | **0.233** | **0** |  |  |  |  |  |  |  |  |  |  |  |  |  |  |  |
|  |  |  | **Asian** | **7** | **4635/4722** | **Allelic** |  | **random** | **0.928 (0.829-1.039)** | **71.4** | **0.196** | **0.002** |  |  |  |  |  |  |  |  |  |  |  |  |  |  |  |
|  |  |  |  |  |  | **Dominant** |  | **random** | **0.950 (0.809-1.115)** | **60** | **0.528** | **0.02** |  |  |  |  |  |  |  |  |  |  |  |  |  |  |  |
|  |  |  |  |  |  | **Recessive** |  | **random** | **0.853 (0.688-1.059)** | **78.2** | **0.149** | **0** |  |  |  |  |  |  |  |  |  |  |  |  |  |  |  |
|  |  |  | **Caucasian** | **1** | **101/129** | **Allelic** |  | **random** | **1.375 (0.934-2.023)** | **Na** | **Na** | **Na** |  |  |  |  |  |  |  |  |  |  |  |  |  |  |  |
|  |  |  |  |  |  | **Dominant** |  | **random** | **1.540 (0.900-2.635)** | **Na** | **Na** | **Na** |  |  |  |  |  |  |  |  |  |  |  |  |  |  |  |
|  |  |  |  |  |  | **Recessive** |  | **random** | **1.604 (0.664-3.880)** | **Na** | **Na** | **Na** |  |  |  |  |  |  |  |  |  |  |  |  |  |  |  |
| **miR-4293** | **rs12220909** | **G>C** | **Asian** | **2** | **1795/1954** | **Allelic** |  | **random** | **0.853 (0.659-1.104)** | **75.3** | **0.227** | **0.044** |  |  |  |  |  |  |  |  |  |  |  |  |  |  |  |
|  |  |  |  |  |  | **Dominant** |  | **random** | **0.799 (0.611-1.046)** | **67.4** | **0.102** | **0.08** |  |  |  |  |  |  |  |  |  |  |  |  |  |  |  |
|  |  |  |  |  |  | **Recessive** |  | **fixed** | **1.031 (0.724-1.466)** | **0** | **0.866** | **0.475** |  |  |  |  |  |  |  |  |  |  |  |  |  |  |  |
| **miR-149** | **rs2292832** | **T>C** | **All** | **3** | **1714/1559** | **Allelic** |  | **fixed** | **0.996 (0.898-1.104)** | **45.8** | **0.935** | **0.158** |  |  |  |  |  |  |  |  |  |  |  |  |  |  |  |
|  |  |  |  |  |  | **Dominant** |  | **fixed** | **1.012 (0.879-1.165)** | **42.4** | **0.87** | **0.176** |  |  |  |  |  |  |  |  |  |  |  |  |  |  |  |
|  |  |  |  |  |  | **Recessive** |  | **fixed** | **0.957 (0.775-1.181)** | **4.3** | **0.681** | **0.352** |  |  |  |  |  |  |  |  |  |  |  |  |  |  |  |
|  |  |  | **Asian** | **2** | **1613/1430** | **Allelic** |  | **fixed** | **1.020 (0.916-1.135)** | **0** | **0.723** | **0.321** |  |  |  |  |  |  |  |  |  |  |  |  |  |  |  |
|  |  |  |  |  |  | **Dominant** |  | **fixed** | **1.035 (0.897-1.195)** | **0** | **0.638** | **0.507** |  |  |  |  |  |  |  |  |  |  |  |  |  |  |  |
|  |  |  |  |  |  | **Recessive** |  | **fixed** | **1.001 (0.795-1.260)** | **17** | **0.996** | **0.272** |  |  |  |  |  |  |  |  |  |  |  |  |  |  |  |
|  |  |  | **Caucasian** | **1** | **101/129** | **Allelic** |  | **fixed** | **0.724 (0.489-1.073)** | **Na** | **Na** | **Na** |  |  |  |  |  |  |  |  |  |  |  |  |  |  |  |
|  |  |  |  |  |  | **Dominant** |  | **fixed** | **0.495 (0.219-1.121)** | **Na** | **Na** | **Na** |  |  |  |  |  |  |  |  |  |  |  |  |  |  |  |
|  |  |  |  |  |  | **Recessive** |  | **fixed** | **0.760 (0.450-1.283)** | **Na** | **Na** | **Na** |  |  |  |  |  |  |  |  |  |  |  |  |  |  |  |
| **miR-499** | **rs3746444** | **A>G** | **All** | **4** | **2359/2364** | **Allelic** |  | **random** | **1.157 (0.951-1.407)** | **65.9** | **0.145** | **0.032** |  |  |  |  |  |  |  |  |  |  |  |  |  |  |  |
|  |  |  |  |  |  | **Dominant** |  | **fixed** | **1.142 (1.009-1.294)** | **55.1** | **0.036** | **0.105** | **ACC** | **Weak** | **1437** | **A** | **C** | **C** |  | **No** | **No** | **Yes** | **0.762** | **0.734** | **No** | **0.415** | **Weak** |
|  |  |  |  |  |  | **Recessive** |  | **fixed** | **1.335 (0.999-1.785)** | **34.8** | **0.051** | **0.203** |  |  |  |  |  |  |  |  |  |  |  |  |  |  |  |
|  |  |  | **Asian** | **3** | **2258/2235** | **Allelic** |  | **random** | **1.186 (0.942-1.493)** | **76.1** | **0.146** | **0.015** |  |  |  |  |  |  |  |  |  |  |  |  |  |  |  |
|  |  |  |  |  |  | **Dominant** |  | **random** | **1.176 (0.936-1.477)** | **67.1** | **0.164** | **0.048** |  |  |  |  |  |  |  |  |  |  |  |  |  |  |  |
|  |  |  |  |  |  | **Recessive** |  | **fixed** | **1.403 (1.034-1.904)** | **42.4** | **0.029** | **0.176** | **BBC** | **Weak** | **177** | **B** | **B** | **C** |  | **No** | **No** | **Yes** | **0.759** | **1** | **No** | **0.459** | **Weak** |
|  |  |  | **Caucasian** | **1** | **101/129** | **Allelic** |  | **random** | **1.005 (0.664-1.520)** | **Na** | **Na** | **Na** |  |  |  |  |  |  |  |  |  |  |  |  |  |  |  |
|  |  |  |  |  |  | **Dominant** |  | **random** | **1.075 (0.638-1.811)** | **Na** | **Na** | **Na** |  |  |  |  |  |  |  |  |  |  |  |  |  |  |  |
|  |  |  |  |  |  | **Recessive** |  | **fixed** | **0.799 (0.298-2.140)** | **Na** | **Na** | **Na** |  |  |  |  |  |  |  |  |  |  |  |  |  |  |  |
| **miR-608** | **rs4919510** | **C>G** | **Asian** | **4** | **2033/2118** | **Allelic** |  | **random** | **1.063 (0.849-1.331)** | **84.2** | **0.596** | **0** |  |  |  |  |  |  |  |  |  |  |  |  |  |  |  |
|  |  |  |  |  |  | **Dominant** |  | **random** | **1.128 (0.896-1.419)** | **63.0** | **0.305** | **0.044** |  |  |  |  |  |  |  |  |  |  |  |  |  |  |  |
|  |  |  |  |  |  | **Recessive** |  | **random** | **1.047 (0.738-1.484)** | **80.6** | **0.797** | **0.001** |  |  |  |  |  |  |  |  |  |  |  |  |  |  |  |
| **AGO1#** | **rs595961** | **A>G** | **Asian** | **2** | **571/492** | **Allelic** |  | **fixed** | **0.692 (0.551-0.869)** | **0** | **0.022** | **0.852** | **AAA** | **Strong** | **1750** | **A** | **A** | **A** |  | **NA** | **No** | **No** | **Na** | **1** | **No** | **0.044** | **Strong** |
|  |  |  |  |  |  | **Dominant** |  | **fixed** | **0.558 (0.245-1.273)** | **35.9** | **0.166** | **0.212** |  |  |  |  |  |  |  |  |  |  |  |  |  |  |  |
|  |  |  |  |  |  | **Recessive** |  | **fixed** | **0.656 (0.506-0.851)** | **0** | **0.002** | **0.482** | **BAA** | **Moderate** | **712** | **B** | **A** | **A** |  | **NA** | **No** | **No** | **Na** | **1** | **No** | **0.059** | **Moderate** |
| **miR-26-1** | **rs7372209** | **T>C** | **Asian** | **2** | **1278/1328** | **Allelic** |  | **fixed** | **0.964 (0.857-1.085)** | **0** | **0.548** | **0.592** |  |  |  |  |  |  |  |  |  |  |  |  |  |  |  |
|  |  |  |  |  |  | **Dominant** |  | **fixed** | **0.937 (0.716-1.226)** | **45** | **0.633** | **0.177** |  |  |  |  |  |  |  |  |  |  |  |  |  |  |  |
|  |  |  |  |  |  | **Recessive** |  | **fixed** | **0.961 (0.824-1.121)** | **43.3** | **0.611** | **0.184** |  |  |  |  |  |  |  |  |  |  |  |  |  |  |  |
| **GEMIN4#** | **rs7813** | **C>T** | **Asian** | **2** | **571/494** | **Allelic** |  | **fixed** | **1.258 (1.050-1.507)** | **62.2** | **0.013** | **0.104** | **ACA** | **Weak** | **1423** | **A** | **C** | **A** | **Na** | **NA** | **No** | **No** | **Na** | **1** | **No** | **0.199** | **Weak** |
|  |  |  |  |  |  | **Dominant** |  | **fixed** | **1.097 (0.756-1.593)** | **0** | **0.625** | **0.988** |  |  |  |  |  |  |  |  |  |  |  |  |  |  |  |
|  |  |  |  |  |  | **Recessive** |  | **random** | **1.227 (0.627-2.403)** | **78.8** | **0.55** | **0.03** |  |  |  |  |  |  |  |  |  |  |  |  |  |  |  |
| **miR-27a** | **rs895819** | **T>C** | **Asian** | **3** | **2123/2191** | **Allelic** |  | **random** | **1.067 (0.903-1.261)** | **66.4** | **0.446** | **0.051** |  |  |  |  |  |  |  |  |  |  |  |  |  |  |  |
|  |  |  |  |  |  | **Dominant** |  | **fixed** | **1.024 (0.908-1.154)** | **37.8** | **0.705** | **0.2** |  |  |  |  |  |  |  |  |  |  |  |  |  |  |  |
|  |  |  |  |  |  | **Recessive** |  | **fixed** | **1.292 (1.041-1.602)** | **48** | **0.02** | **0.146** | **BBC** | **Weak** | **371** | **B** | **B** | **C** | **HWE** | **No** | **No** | **Yes** | **0.565** | **1** | **No** | **0.289** | **Weak** |
| **GEMIN4#** | **rs910924** | **C>T** | **Asian** | **2** | **566/485** | **Allelic** |  | **fixed** | **0.732 (0.570-0.940)** | **0** | **0.015** | **0.35** | **BAA** | **Moderate** | **284** | **B** | **A** | **A** |  | **No** | **No** | **No** | **Na** | **1** | **No** | **0.264** | **Weak** |
|  |  |  |  |  |  | **Dominant** |  | **fixed** | **0.704 (0.532-0.931)** | **0** | **0.014** | **0.336** | **BAA** | **Moderate** | **264** | **B** | **A** | **A** |  | **No** | **No** | **No** | **Na** | **1** | **No** | **0.288** | **Weak** |
|  |  |  |  |  |  | **Recessive** |  | **fixed** | **0.689 (0.283-1.676)** | **0** | **0.411** | **0.801** |  |  |  |  |  |  |  |  |  |  |  |  |  |  |  |

Note: A, adenine; C, cytosine; G, guanine; T, thymine; OR, odds ratio; CI, confidence interval; MAF, minor allelic frequency in control; NA, not applicable; FPRP, false positive report probability.

^1^allelics: Minor allelic (bold) versus major allelic.

^2^Venice criteria grades are for amount of evidence, replication of the association and protection from bias.

^3^The prior probability of FPRP is 0.05, and the FPRP level of noteworthiness is 0.20.

^#^microRNA (miRNA) biosynthesis genes
