# Supplementary material for: Epidemiological evidence for associations between variants in microRNA or biosynthesis genes and lung cancer risk
Source: Cancer Med. 2020 Jan 7;9(5):1937–50. doi: 10.1002/cam4.2645 (PMC7050065; doi:10.1002/cam4.2645)
Supplement: Supplementary file 16 [file CAM4-9-1937-s016.docx]

**Supporting information to Notes for Venice Criteria**

We applied the Venice Criteria to evaluate the epidemiological credibility of significant associations identified by meta-analysis. Briefly, credibility was defined as strong, moderate, or weak, based on the grade of A, B, or C in three categories: amount of evidence, replication of the association, and protection from bias.

**Amount of evidence**

A: Large-scale evidence — minor genetic group (alleles or genotypes) in cases and controls > 1,000.

B: Moderate amount of evidence — minor genetic group in cases and controls between 100 and 1,000.

C: Little evidence — minor genetic group in cases and controls < 100.

**Replication of association**

A: Little between-study heterogeneity — *I*^2^ < 25%.

B: Moderate between-study heterogeneity — *I*^2^ between 25% and 50%.

C: Large between-study heterogeneity — *I*^2^ > 50%.

Qualitative epidemiologic considerations about the presence of heterogeneity and potential explanation for heterogeneity would need to be taken into account in judging replication. It may be reasonable to grade as A on this criterion for associations with moderate or high heterogeneity with an extensive replication record such as associations identified by GWAS or large GWAS meta-analysis from collaborative studies.

**Protection from bias**

A: No observable bias and bias was unlikely to explain the presence of the association. B: No obvious bias may affect the presence of the association, but there is considerable missing information on the identification of evidence. C: Bias is demonstrable or is likely to explain the presence of the association. The Venice criteria include an extensive checklist for sources of bias in different settings. The checklist has different considerations depending on whether the evidence comes from retrospective meta-analyses of published data or prospective GWAS and replication studies from collaborative consortia with harmonization of data collection and analysis.

General checks for bias that have been adopted for meta-analysis are: (1) Association lost with exclusion of first study; (2) Association lost with exclusion of studies deviated from HWE; (3) Small effect size of association (i.e., 0.87 < OR < 1.15); (4) Evidence of publication bias (*p* < 0.10 in Begg's test); (5) Evidence of small-study effect (*p* < 0.10 in Egger's test); (6) Evidence is presented for an excess of individual studies with significant findings (*p* < 0.10 in significant bias test).
